# Asian Best Practices for Care of Diabetes in Elderly (ABCDE)

**DOI:** 10.1900/RDS.2022.18.100

**Published:** 2022-06-30

**Authors:** Sanjay Kalra, Minakshi Dhar, Faria Afsana, Pankaj Aggarwal, Than Than Aye, Ganapathy Bantwal, Manash Barua, Saptarshi Bhattacharya, Ashok Kumar Das, Sambit Das, Arundhati Dasgupta, Guruprasad Dhakal, Atul Dhingra, Fatemeh Esfahanian, Sharvil Gadve, Jubbin Jacob, Nitin Kapoor, Ali Latheef, Yovan Mahadeb, Robin Maskey, Wali Naseri, Jeya Ratnasingam, Abbas Raza, Banshi Saboo, Rakesh Sahay, Mona Shah, Shehla Shaikh, SK Sharma, Dina Shrestha, Noel Somasundaram, Mangesh Tiwaskar, Apurva Jawdekar

**Affiliations:** 1 Bharti Hospital, Karnal, India,; 2 AIIMS Rishikesh, India,; 3 BIRDEM, Bangladesh India,; 4 Hormone Care & Research Center, India,; 5 Grand Hantha International Hospital, Myanmar,; 6 St Johns Medical College & Hospital, India,; 7 Excel Care Hospitals, India,; 8 Max Super Specialty Hospital, India,; 9 Pondicherry Institute of Medical Science (PIMS), India,; 10 Dr.Sambit’s Centre of Diabetes and Endocrinology, India,; 11 Rudraksh Super Specialty Care, India,; 12 Faculty of Postgraduate Medicine, Bhutan,; 13 Gangaram Bansal Hospital, Sri Ganganagar, India,; 14 Tehran University of Medical Sciences, Iran,; 15 Excel Endocrine Centre, India,; 16 Christian Medical College Ludhiana, India,; 17 Christian Medical College, Vellore, India,; 18 National Diabetes Centre, Indira Gandhi Memorial, India,; 19 Bruno Cheong Hospital, Mauritius,; 20 BPKIHS, Nepal,; 21 Kabul Medical University, Afghanistan,; 22 University of Malaya, Malaysia,; 23 Shaukat Khanum Cancer Hospital and Research Centre, Pakistan,; 24 Diabetes Care and Hormone Clinic, Ahmedabad, India,; 25 Osmania Medical College & Hospital, India,; 26 Sterling Hospital, India,; 27 Saifee Hospital, Mumbai, India,; 28 Galaxy Specialty Centre, India,; 29 Norvic International Hospital, Nepal,; 30 Nawaloka Hospitals, Sri Lanka,; 31 Shilpa Medical Research Centre, Mumbai, India,; 32 Lupin Ltd, India..

**Keywords:** type 2 diabetes, elderly, geriatric syndrome, hypoglycemia, falls, therapeutic parsimony, technological aids, pharmacological and non-pharmacological interventions

## Abstract

The elderly population with diabetes is diverse with the majority experiencing a decline in physical and mental capabilities, impacting the entire diabetes management process. Therefore, a need for geriatric-specific guidelines, especially for the Asian population, was identified and subsequently developed by an expert panel across government and private institutions from several Asian countries. The panel considered clinical evidence (landmark trials, position papers, expert opinions), recommendations from several important societies along with their decades of clinical experience and expertise, while meticulously devising thorough geriatric-specific tailored management strategies. The creation of the ABCDE best practices document underscores and explores the gaps and challenges and determines optimal methods for diabetes management of the elderly population in the Asian region.

## 1 Introduction

The elderly population with diabetes is diverse. Some have physical and mental capacities like many younger ones, whereas others experience considerable declines in these capabilities. This impacts the entire diabetes management process. Therefore, it is imperative to address the differences in managing diabetes in the elderly population in a patient-centric manner. To obtain multiple perspectives in this regard, the Asian hybrid steering committee meeting on geriatric diabetes was conducted in Rishikesh, India. The expert panel involved key opinion leaders and representatives of the government as well as private institutions from Afghanistan, Bangladesh, Bhutan, India, Iran, Malaysia, Maldives, Mauritius, Myanmar, Nepal, Pakistan, and Sri Lanka.

The participants deliberated on a range of aspects of diabetes, clinical evidence (landmark trials, position papers, expert opinions), and recommendations of the American Diabetes Association (ADA), American Association of Clinical Endocrinology (AACE), European Association for the Study of Diabetes (EASD), International Diabetes Federation (IDF), Diabetes Canada, European Society of Endocrinology (ESE), the Gerontological Society of America (GSA), and The Obesity Society (TOS) in the management of the elderly population. They exchanged their peculiar understandings, experiences, and insights regarding the management of elderly persons with diabetes to develop tailored care for elderly diabetics.

The integration of technological aids, and environmental modulation to facilitate the management of diabetes in the presence of problems unique to the elderly was elaborately discussed and best practices were agreed upon. The consensus was drafted on these principles and circulated among expert panel members for their critical appraisal; it was subsequently amended with appropriate changes. Thus, the aim of the ABCDE to underscore and explore the gaps and challenges and determine optimal methods in diabetes management of the elderly population in the Asian region was achieved.

### 1.1 Elderly in Asia

#### 1.1.1 Definition of ‘Geriatric

The term “geriatric” has had different definitions over the past decades. A few decades ago, those ≥ 85 years of age were classified as oldest-old. Later, older adults were classified into three groups young-old (65-74 years of age), middle-old (75-85 years of age), and the oldest-old (> 85 years of age). Whereas World Health Organization (WHO) defines older persons as ≥ 60 years of age, most countries usually consider 60-65 years of age as geriatric [1,2]. However, for clinical purposes, rather than classifying older adults based on chronological age, a classification based on functional capabilities is more pragmatic. The functional aspects are influenced by genetics, environmental features, and co-morbidities such as metabolic disorders, hypertension, arthritis, obesity, renal function, and most importantly, cognitive abilities, which indeed reflect the physiological and vascular age. Thus, based on the International Diabetes Federation (IDF) classification the ABCDE experts have appropriately categorized older adults into four groups/categories based on functional capacities, level of medical comorbidities, and degree of frailty, which should guide diabetes management **(Table 1)** [3].

**Table 1.** Functional classification of older adults

Category	Functional group	Disease conditions	Degree of frailty
I	Functionally independent and self-reliant	Only Diabetes or non-life-threatening disease conditions	Not frail
II	Frail patients without cognitive impairment	Suffer from fatigue, weight loss, and extremely restricted	Frail
	Partially dependent on others	mobility and/or strength; highly susceptible to falls and institutionalization	
III	Frail patients with cognitive impairment/	Increased risk of poor control leading to glycemic extremes	Frail
	dementia, completely dependent on caregivers	(hypoglycemia and hyperglycemia)	
IV	Terminally ill patients	Significant co-morbidities or malignancy, short lifespan	Extremely Frail

### 1.2 Prevalence of Type 2 Diabetes Mellitus in Asia

Globally, there were 704 million older adults (65-99 years) in 2019, a number that is projected to increase to 995 million in 2030 and 1.4 billion by 2045. The estimated prevalence of diabetes in this population seems constant ranging from 19.3% in 2019 to 19.6% in 2045. Nevertheless, the estimated number of elderly with diabetes will show a considerable spike by 2045 (276.2 million) compared to 2019 (135.6 million), which is more than twice that in 2019, if the trend continues. Thus, diabetes prevalence increases with age so the highest estimated prevalence is in adults > 65 years of age.

The past few decades have seen a rise in the number of persons with type 2 diabetes, with more than 60% of these living in Asia (approximately 230 million with an estimated rise above 350 million by 2040) [4]. The South Asian countries will see a 150% rise from 2000 to 2035. According to the Diabetes Atlas 2021, Southeast Asia has 90 million diabetics [5]. Among older adults (≥ 65 years) it is estimated that diabetes will double from 35.5 million in 2019 to more than 78 million in 2045 [5,6]. India and Pakistan are ranked among the top ten countries with elderly persons living with diabetes and would jump from third to second place and tenth to seventh place by 2045 diabetic patients globally [5,7]. India is projected to have 27.5 million elderly persons living with diabetes in 2045 compared to 12.1 million in 2019, and thus, would replace the United States (23.2 million) for the second position globally. Pakistan is estimated to have 6 million compared to 2.6 million for the same years, also scaling up from tenth to the seventh position, globally. Among the SEA countries, India and Pakistan would retain their top two positions in 2030, followed by Bangladesh with more than 2 million elderlies living with diabetes. In Singapore, a local survey found that 29.1% between 60 and 69 years old have diabetes. It also predicted that one in two Singaporeans will experience diabetes by the age of 70 [8]. The prevalence of elderly persons with diabetes in Thailand was 17.2% as per the InterASIA 2003 study [9]. The prevalence of diabetes was ~11%-13% in the Vietnamese population over the age of 60 years [10]. According to the World Diabetes Federation atlas, the number of elderly with diabetes would increase by more than 200% in two decades in the majority of Asian countries. Thus, these data indicate an enormous surge in the aging population with diabetes in the subsequent decades.

The South Asian region is unique as it still faces the dual pandemic of managing undernourishment and infective disorders at one end and has a rapidly increasing burden of non-communicable disorders like diabetes on the other end of the spectrum. This was further compounded by the COVID-19 pandemic [11,12]. Moreover, the unique thin-fat phenotype which is seen in the South Asian region makes the elderly more prone to develop diabetes with a lower body mass index [13,14]. Furthermore, the dietary pattern in the South Asian region, is a carbohydrate predominant non-diabetic friendly eating pattern, which is difficult to change in an older person [15]. This, along with lower per capita income in the elderly and increasing costs of treatment, in a genetically prone ethnicity, makes diabetes in the elderly even more challenging in this region [16,17]. Thus, the rapidly increasing diabetes burden coupled with the unique features of a thin-fat phenotype, high carbohydrate staple diet, poor affordability, and high-risk ethnicity makes the South Asian population a challenging one to manage. This foreseeable health threat prompted the experts across these countries to develop a consensus addressing the unique aspects of diabetes in older adults, particularly tailoring intervention according to functional status and co-morbidities.

### 1.3 Physiology

**Figure 1.**
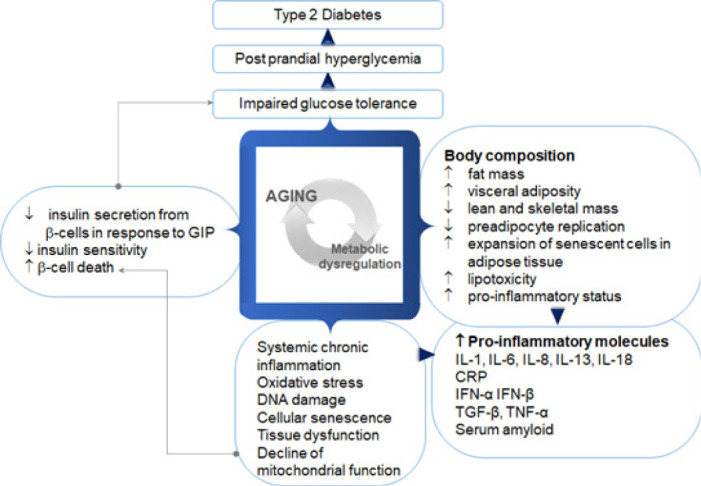
Aging and type 2 diabetes: Aging worsens systemic inflammation, increases oxidative stress, and poor mitochondrial function resulting in metabolic abnormalities. It also alters body composition predisposing older adults to develop visceral adiposity further inducing pro-inflammatory mediators and amplifying systemic inflammation. It also impacts the beta-cell physiology culminating in impaired responses to endogenous incretins, thus accelerating the development of clinical diabetes manifestations [18].

Chronological age itself is an independent risk factor for diabetes as well as other chronic diseases [18,19]. Diabetes mellitus is a precipitant of aging. Thus, common pathways partake in the pathophysiology of diabetes and aging. Numerous factors contribute to the pathophysiology of diabetes in older age (Figure 1).

Regulation of insulin secretion in response to blood glucose levels permits maintenance of normal range. Unlike young adults, older adults become progressively incapable of regulating glucose metabolism. This is especially true in the post-absorptive phase during which normally half of the hepatic glucose is used by the brain and approximately one-seventh by the skeletal muscle [19].

The Baltimore Longitudinal Study of Aging conducted twice in a span of 30 years found that the impaired response to the two-hour OGTT had a positive association with age; though the glucose tolerance deteriorated from the fourth decade up to the ninth decade of life, the alteration from the sixth decade onwards remained significant even after adjusting for BMI, unlike up to the fifth decade. These findings indicate that up to the fifth decade, blood glucose abnormalities are influenced by body fat and physical activity. With advancing age, plasma glucose levels in response to OGTT were progressively higher for every decade of age until they peaked at the seventh decade, more so in males than females. Although the FPG was attenuated after adjusting for BMI, the correlation of 2hG with age remained significant [19].

On the other hand, studies also confirmed that body composition rather than age (across 30-90 years) affects hepatic glucose production [19]. The association between age and insulin resistance is ambiguous. The insulin-glucose dose-response curve shifts to the right with age both in single- and multiple-insulin dose studies. These data mean that compared to the young, for the same amount of glucose, twice the amount of insulin is required in the old. This association remained significant after adjusting for BMI [19]. When adjusted for body composition, physical activity, and peripheral insulin sensitivity, the effect of age on the non-insulin mediated peripheral glucose uptake is obscure [19]. Aging may reduce the amplitude and mass of rapid insulin pulses during fasted and fed state; although a reduction in the frequency of ultradian pulses occurs during the fasted state and it is regularized in the fed state in the old, its clinical relevance remains unknown [19].

Studies that evaluated the effect of age on β-cell function using hyperglycemic clamps found that the second phase of insulin responses reaches a plateau in nearly half the duration in the elderly than in the young (120-150 minutes vs. 300 minutes) [19]. Incretins are enteroendocrine hormones (glucose-dependent insulinotropic polypeptide (GIP) and glucagon-like peptide-1 (GLP-1) that enhance insulin response from β-cells beyond the amount ascribed to glucose alone [19].

Using a hyperglycemic clamp, in combination with OGTT studies that examined the effect of age on the sensitivity of β-cells to endogenous GIP, researchers found that β-cell sensitivity to GIP is reduced in advancing age. The insulinotropic effect of GIP diminishes with age but is unaffected at very high plasma glucose levels. These suggest that the age-related impairment in response to GIP is a significant cause of glucose intolerance [19].

Insulin resistance is primarily attributed to visceral obesity. Overconsumption of food leads to accelerated weight gain in older compared to younger persons because aging is associated with gradual loss of muscle mass and gain in fat mass from the fourth decade. Menopause in women and decline in testosterone with age in men aggravates the loss of lean muscle mass and increase in adipose tissue with suppressed catecholamine-induced lipolysis [19].

### 1.4 Alzheimer’s disease and diabetes

Alzheimer’s disease (AD) and type 2 diabetes mellitus are two of the most widespread diseases in the elderly population globally. Pathologically, AD is characterized by extracellular plaques of amyloid-β and intracellular neurofibrillary tangles of hyperphosphorylated tau [20]. Type 2 diabetes is a metabolic disorder marked by hyperglycemia, insulin resistance, and the formation of human islet amyloid polypeptide that causes pancreatic β-cell dysfunction [20]. The term “type 3 diabetes” may echo the plausibility that Alzheimer’s disease denotes a form of diabetes that only comprises the brain. Both tend to have certain common molecular and biochemical features including, but not limited to, insulin deficiency, insulin resistance, impaired energy metabolism, mitochondrial dysfunction, and oxidative stress [21].

### 1.5 Screening and diagnosis

The recent ADA, AACE, and United States Preventive Services Task Force (USPSTF) 2021 recommend widespread screening for prediabetes and diabetes, using FPG, 2h-OGTT, or A1C level, for all adults ≥ 45 years, regardless of risk factors, and screening adults who are overweight or obese (BMI ≥ 25 or ≥ 23 in Asian Americans) with one or more risk factors, regardless of age. If the glycemic indices are normal, repeat screening is recommended at a minimum of three-year intervals. However, the endocrine societies, namely the ESE, the GSA, and TOS differ from the recommendations of the diabetes associations. For patients aged ≥ 65 years without known diabetes, only the measurement of A1c may be erroneous. This is attributed due to potential comorbidities that can affect the lifecycle of red blood cells in elderly persons. Hence, these guidelines recommend that older persons with normal glycemic indices undergo repeat screening every two years; subject to the prospective benefit, terminally ill patients with malignancy or organ system failure, therefore, could be exceptions. In patients ≥ 65 years of age with abnormal FPG or A1c, a 2h-OGTT could be useful in high-risk patients (overweight or obese, first-degree relative with diabetes, Asians, history of hypertension, dyslipidemia, sleep apnea, or physical inactivity). In this high-risk group, shared decision-making is suggested if a patient is frail or finds the investigation procedure excessively onerous [22]. Nevertheless, 2h-OGTT rises by 5-10mg/ dL and FPG 1–2 mg/dl every subsequent decade and may be prone to overdiagnosis.

**‘**The experts in the current consensus agree with the Endocrinology Societies’ recommendations in that A1c alone may be inadequate. However, routine screening with FPG alone may also lead to underdiagnosis, hence the ABCDE suggests 2h-OGTT as a better diagnostic marker in the elderly as substantiated by the data from BLSA.**’**

The BLSA found that if only FPG and A1c are used for diagnosis, more than one-third of those with diabetes mellitus would be missed, particularly Asians with low A1c (because it is influenced by ethnicity) [23]. Therefore, it may be agreed that postprandial hyperglycemia is an important feature of type 2 diabetes in the older population and an OGTT is essential to avoid misdiagnosis based on normal FPG or A1C alone [18]. Hence, the ABCDE recommends OGTT for ruling out diabetes in at least high-risk groups.

**‘**The ABCDE recommends screening at the available timepoints along with other routine investigations in the elderly and routine annual screening in older adults with risk factors, particularly high BMI (overweight/ obese), hypertension, dyslipidemia, and heart disease.**’**

Screening for diabetes not only reduces morbidity but also considerably helps to diminish the risk or severity of associated complications. Among more than 150,000 postmenopausal women, the Women’s Health Initiative study found that severity of vasomotor symptoms (night sweats and hot flashes) was proportionally associated with increased risk of diabetes (mild VMS 13%, moderate VMS 29%, severe VMS 48%) irrespective of obesity [24].

**‘**The ABCDE experts believe that menopause transition may be an ideal window for clinicians to screen for diabetes in women.**’**

In the Asian geriatric population, a meta-analysis revealed that the prevalence of sarcopenia is higher in persons with diabetes than without (15.9% vs. 10.8%); therefore, screening for diabetes is relevant [25].

A study from India reported diabetes as a risk factor for dementia (33.3% of those with diabetes vs. 9% of those without) [26]. Studies from India also have suggested that the ApoE4 allele is significantly associated with AD. Other reports from Asia confirm the association between type 2 diabetes and AD in ApoE4 carriers. A meta-analysis of 28 prospective observational studies found that diabetes increased the risk of all types of dementia by 73% and AD by 56% compared to people without diabetes [27,28]. Therefore, persons diagnosed with dementia should be screened for diabetes.

**‘**The ABCDE experts opine those persons with sarcopenia and dementia should be screened for diabetes.**’**

### 1.6 Clinical features

#### 1.6.1 Unique Symptoms

Elderly persons may present with atypical symptoms, and thus, may delay the recognition of diabetes **(Table 2)**. The subtleness, atypical or delayed manifestation of the classical symptoms are misleading. The elevated renal threshold for glucose excretion and diminished thirst perception alters the presentation of classical symptoms [29-32].

**Table 2.** Elderly persons with diabetes: a unique presentation

Subtle	Predominant
Classical/specific symptoms	**Non-classical/Non-specific symptoms**
Polyuria/Nocturia Hyperphagia Polydipsia	Dry mouth Incontinence Dehydration Confusion Unexplained weight loss Fatigue Lethargy Frequent falls Skin Infections Genitourinar y Infections

#### 1.6.2 Traditional Vascular Complications

Older persons with diabetes have poorer end-organ function due to aging and co-morbidities. One or more co morbidities are present in 60% and ≥ 3 co-morbidities are present in 40% of the elderly persons with diabetes [18,33].

#### 1.6.3 Macrovascular (Cardiovascular, Cerebrovascular, Peripheral vascular Disease) Complications

More than 40% of elderly adults with diabetes experience some type of coronary heart disease (CHD) [34]. More than 20% of older adults with diabetes have silent myocardial ischemia [35]. Among patients aged 65-84 years, the prevalence of MI was higher in those with than without diabetes (11.3% vs. 8%; p = .032). The CV health study in adults aged ≥ 65 years showed that the CHD-associated mortality in persons with diabetes either treated with oral agents or insulin was more than twice as high as those without [36]. Thus, among older patients with diabetes, CHD is a leading cause of fatal outcomes. Like CHD, the prevalence of CVD in older adults aged between 65 to 84 years is significantly higher in those with than without diabetes (10.6% vs. 7%; p = .003) [37]. Advancing older age intensifies the risk of developing PVD. This was corroborated by an Indonesian study in which persons with type 2 diabetes in the seventh decade were sevenfold more likely to develop PVD compared to those in the sixth decade of life [38].

#### 1.6.4 Microvascular (Retinopathy, nephropathy, neuropathy) Complications

Unlike the macrovascular, the prevalence of ocular complications of diabetes in the elderly (≥ 65 years) is similar to that in middle-aged (40-64 years) patients (29.5% vs. 28%; p = .64) [39]. Moreover, retinopathy is less common among adults diagnosed with diabetes in older age compared to middle age owing to the lesser duration of disease [39].

In older adults (> 60 years), the most common basis for CKD and end-stage renal disease (ESRD) is diabetic nephropathy [40]. The prevalence of CKD was significantly higher in individuals > 65 years diagnosed with diabetes compared to those without, with an absolute difference of 8%-17% (Kidney Early Evaluation Program 48.2% vs. 40.4%, NHANES 58.3% vs. 41.4%, Medicare 14.2% vs. 4.4%; p < .001) [41].

Similar to CV complications, long-standing (≥ 25-year) diabetes increases the risk of neuropathy with more than 50% being diagnosed with diabetic peripheral neuropathy (DPN) [42-44]. The presence of DPN may impair their balance due to malfunctioning of the three major elements: sensory (lack of motion), motor (impaired movement coordination), and autonomic (the existence of postural hypotension) [45]. Diabetic peripheral neuropathy increases the risk of falls almost five-fold.

Further autonomic neuropathy is suspected to be a modifier of the extent of QT-prolongation which is a known risk factor for ventricular arrhythmias [46]. The Action to Control Cardiovascular Risk in Diabetes (ACCORD) trial found a higher incidence of hypoglycemia and mortality in intensively treated type 2 diabetes patients. Thus, it is plausible that hypoglycemia induces cardiac arrhythmias in patients with type 2 diabetes, causing the ‘dead-in-bed syndrome’ [46].

### 1.7 Geriatric syndromes and Diabetes: Geriatric-specific Complications

In addition to the traditional vascular complications which affect the quality of life, the incidence of a set of conditions termed geriatric syndromes, prevails at a greater frequency in older adults with diabetes which leads to diminished self-care abilities and clinical outcomes.

#### 1.7.1 Menopause

##### Prevalence

The proportion of older adults/elderly and those diagnosed with diabetes is progressively increasing, and therefore, menopausal women may form a substantial proportion of the diabetic population (19.4% post-menopausal vs. 12.1% pre-menopausal in Asian women) [47].

##### Pathophysiology

Menopause has deleterious effects on body weight and adipose tissue distribution. Further reduced energy expenditure along with diminished insulin secretion and decreased insulin sensitivity increase the risk of diabetes in addition to aging. The surge in visceral obesity-associated pro-inflammatory state and androgen excess creates an insulin-resistant environment which increases the incidence of metabolic syndrome compared to reproductive age women (30%-70% vs. 14%-45%). However, data obtained from the Women’s Health Study have shown that glucose abnormalities observed during menopause are associated with age and not with the waning ovarian function [48]. On the contrary, several studies have shown that premature menopause, whether spontaneous or induced or lesser number of reproductive years, is associated with a 20%-57% increased risk of diabetes, with some studies reporting a risk of 18% after adjusting for obesity [24,49-52]. Diabetes per se may hasten the arrival of menopause [53].

##### Treatment

The basis of managing menopausal diabetic women is lifestyle intervention involving a diet with an energy deficit of 500-750 kcal/day and ≥ 150 minutes/week of moderate exercise or ≥ 75 minutes/week of vigorous exercise [54,55]. Gradual weight loss (NMT 5%–7% of initial body weight per annum *)* is recommended; since declining, bone mineral density and sarcopenia are important concerns in postmenopausal women [56,57]. Smoking cessation is recommended because it is a known risk factor for CVD and osteoporosis similar to diabetes and aging [57].

Compensatory estrogen supplementation (hormone replacement therapy-HRT) may improve glucose metabolism regardless of diabetes status. In women with pre-existing diabetes and negligible CVD risk, oral oestrogens may be chosen, whereas, in postmenopausal obese and diabetic women, transdermal 17 β-oestradiol should be preferred. Notwithstanding, a progestogen such as progesterone or transdermal norethisterone should be chosen to prevent effects on glucose metabolism.

In women on HRT, the incidence of diabetes reduced from 21% to 12% owing to the improvement in adiposity-associated changes and pro-inflammatory state. But HRT is not a chronic therapy and evidence substantiating its use for diabetes prevention is weak [58]. However, in diabetic women who attain menopause, HRT enhances glycemic control and thereby lowers the dosages of OHA; however, evidence confirming the effect on CVD is missing. Possibly, HRT may be useful in diabetic women during the early menopausal period unlike in older women in whom destabilization of atheroma may occur resulting in acute thrombotic episodes [57].

Studies also have found a higher incidence of urinary tract infections (UTIs) in postmenopausal women with type 2 diabetes [59]. Use of estrogen, either oral or vaginal, maintenance of healthy vaginal flora using vaginal formulations, and antimicrobial therapy may be useful in the prevention and treatment of UTIs in this patient population [59].

**‘T**he ABCDE experts recommend an evaluation of preexisting CVD to determine the benefit of HRT regardless of a diabetes diagnosis. Lifestyle intervention involves diet and moderate/vigorous exercise [54,55]. Gradual weight loss (NMT 5–7% of initial body weight per annum) [56,57]. Smoking cessation is recommended [57].**’**

#### 1.7.2 Late-Onset Hypogonadism in Men

##### Prevalence

Late-onset hypogonadism (LOH) or age-associated testosterone deficiency syndrome occurs with advancing age [60]. Hypogonadism is reported in 33%-64% of men with diabetes and men with low testosterone levels are at risk of impaired glucose tolerance [61-63,65].

##### Pathophysiology

The association between LOH and diabetes is bidirectional/two-way; insulin resistance is a shared feature [64]. Insulin resistance is both the horse and cart in hypogonadism [65]. This association may be true because high testosterone levels have shown a 42% reduction in the risk of development of diabetes [65-69]. The concern in patients with diabetes and LOH is that both may additively increase the risk of fractures [64]. Male patients with both conditions have high or normal BMD; however, the bone dimensions are lower, with a significantly decreased bone turnover, which is primarily the predominant effect of diabetes on bone. Thus, reduced osteoblastogenesis and osteoblast activity could be decisive elements affecting bone health in males with LOH and diabetes [65].

##### Treatment

Although few patients may desire relief from hot flashes, increased sweating, loss of libido, low energy levels, and erectile dysfunction, others may be keen on improving bone health and muscle mass. It is necessary to contemplate those symptoms such as fatiguability, sarcopenia, loss of bone mass, central obesity, and sexual dysfunction that are common to LOH and diabetes. Hence lifestyle intervention is essential along with HRT to enhance its effectiveness. Sometime, nonpharmacological means may even preclude the use of hormone replacement.

Observational studies and systematic reviews have demonstrated the benefits of testosterone replacement therapy (TRT) in hypogonadal men with diabetes. The TIMES2 study showed beneficial effects of TRT on insulin resistance, total and LDL-cholesterol, lipoprotein-a, and sexual health.

Individualized decision-making based on concurred and symptom-led outcomes enhances adherence and improved the probability of attaining endpoints [70].

‘Experts recommend, an individualized and patient-centric approach as it is pragmatic and rationale and enhances compliance since the expectation of patients are met [70]. Only TRT may not be adequate, lifestyle intervention may enhance its effectiveness and sometimes even preclude its need’

#### 1.7.3 Sarcopenia

The Asian Working Group for Sarcopenia (AWGS) defines it as low muscle strength, low muscle mass, and poor physical performance measured as handgrip strength: < 26 kg (men) and < 18 kg (women), ALM/ height^2^: < 7.0 kg/m^2^ (men) < 5.4 kg/m^2^ (women) and gait speed ≤ 0.8 m/s (6-m course), respectively [71].

##### Prevalence

According to the AWGS criteria, the prevalence of sarcopenia in Asian adults (aged ≥ 65 and > 60 years, respectively) with type 2 diabetes is 15% using the AWGS definition [72,73]. Older adults with sarcopenia may have an increased risk of developing type 2 diabetes as confirmed in large epidemiological studies which showed the double risk of developing diabetes in individuals in the lowest skeletal muscle mass quartile compared to those in the highest quartile [74-76]. Lower handgrip strength was also prognostic of higher fasting glucose in subjects over 40 years [77]. The Korean Sarcopenic Obesity Study, Health ABC Study, and the English Longitudinal Study of Ageing demonstrated that patients with type 2 diabetes have three times the risk of low skeletal muscle mass, diminishing muscle mass over five years, and 43% greater odds of low handgrip strength at the end of eight years compared to non-diabetic controls [78-80].

##### Pathophysiology

Sarcopenia similar to LOH is the cause and result of diabetes (bidirectional), predisposing older adults to the development and worsening of diabetes through abnormal glucose clearance due to low muscle mass (normally responsible for 80% glucose disposal), and also increased inflammation due to cytokines produced by the excess adipose tissue [81]. Insulin resistance, inflammation, AGE buildup, augmented oxidative stress, and vascular complications impact muscle health; and weakened muscle health also enhances the risk of developing and worsening type 2 diabetes [82].

##### Treatment

Exercise and dietary changes, especially improving the dietary protein intake are the cornerstones of therapy for patients with diabetes and sarcopenia [82]. Resistance training is the most effective strategy for improving lean muscle mass, strength, and metabolic health in sarcopenic and diabetic individuals. It also reduces the muscle loss associated with caloric restrictions by 50% [83]. Aerobic exercise improves mitochondria-derived oxidative stress and may at least partially benefit persons with sarcopenia [84]. However, their efficacy in older diabetic and sarcopenic adults is yet uncharted. Furthermore, improving dietary protein is also challenging in the South Asian region.

There are several drug classes in the pipeline which may improve muscle mass, and physical function, thus improving the metabolic status of diabetes patients [73].

‘Recommendations: The experts, suggest a balance of dietary modifications, aerobic and anaerobic exercise in older diabetic adults at risk of sarcopenia. In the functional elderly individuals, resistance training should be encouraged.’

#### 1.7.4 Osteoporosis

##### Prevalence

Advancing age and lifestyle, both affect the incidence of type 2 diabetes and osteoporosis and both frequently co-occur. Type 2 diabetes mellitus is a significant risk factor for fractures. Though the Bone mineral density (BMD) may be normal in these persons with type 2 diabetes, the fracture risk is increased, indicating deteriorating bone health.

##### Pathophysiology

Type 2 diabetes directly affects bone metabolism and strength, certain anti-diabetic medications affect bone metabolism, and there is an association between diabetic complications and risk for falls and subsequent fractures [56]. Long-standing diabetes, its poor control, and associated complications have been shown to increase the risk of fractures [85-89]. Although high FPG is associated with an increased risk of fracture, elevated PPG has shown the reverse which could be due to high BMI [56]. Furthermore, unlike type 1, type 2 diabetes is not a listed secondary cause of osteoporosis in the FRAX online tool which calculates the ten-year fracture risk probability since BMD is usually higher in those with than without diabetes [56]. An increased risk of hip fracture is observed even in persons with higher BMD. Clinical evidence in older adults suggests that femoral neck BMD T-score and the WHO FRAX score were associated with hip and nonvertebral fractures. Compared to those without diabetes, the fracture risk was higher in persons with diabetes for a given T-score, FRAX score, and age [90]. Bone mineral density assessment as a standalone tool may not sufficiently represent bone health in persons with diabetes [91]. Therefore, instead of BMD, the trabecular bone score related to bone microarchitecture and fracture risk which provides information independent of BMD should be preferred in elderly with diabetes and post-menopausal women [91,92].

Hyperglycemia, oxidative stress, and the formation of AGEs, negatively affect bone remodeling. Vitamin D deficiency, which is found in diabetics due to various interrelated factors such as obesity, less physical activity, and reduced sun exposure, aggravates calcium deficiency and may increase the risk of osteoporosis.

##### Treatment

As per the Indian Society for Bone and Mineral Research (ISBMR) position statement, in elderly individuals with type 2 diabetes, the intervention threshold should be increased to T-score ≤ −2.0 at the femoral neck or total hip or lumbar spine measured by DXA, unlike non-diabetics in whom T-score ≤ -2.5 [93].

Appropriate exercise program after evaluation of the multifactorial fall risk assessment.

Selective use of orthotics could help reduce discomfort, prevent falls and fractures, and improve quality of life. Supplementation with vitamin D3 greater than 1000 to 2000 international units (IU) of daily maintenance therapy is recommended to maintain an optimal serum 25(OH)D level [93]. Adequate dietary intake of calcium with a total intake (including diet plus supplement, if needed) of ≥ 1000 mg/day is recommended. Bisphosphonates, raloxifene, denosumab, and teriparatide increase BMD in patients with diabetes, the former reduce the risk of vertebral and latter non-vertebral fractures [56,93-95].

‘The trabecular bone score related to bone microarchitecture and fracture risk should be used for evaluation of osteoporosis in elderly persons and post-menopausal women with diabetes, instead of BMD. In suspected cases of secondary osteoporosis, FPG, PPBG, and A1C should be evaluated in a known or suspected case of Type 2 diabetes.’

#### 1.7.5 Impaired Cognition and Dementia

Aging is an established risk factor for cognitive impairment and dementia, and co-existing diabetes worsens it [96].

##### Prevalence

A pooled analysis of 14 studies across Asia, Europe, and the USA found that diabetes was significantly correlated with ~ 60% increased risk of dementia, and there was a 40% risk of nonvascular dementia [97]. More than 66,000 elderly (≥ 65 years) patients with diabetes were found to have a 28% greater risk of cognitive impairment compared to those without in a cross-sectional study using the Abbreviated Mental Test [98]. The prevalence progressively increased with increasing age; from 13.1% for the age group 65-74 years to 24.1% in those > 75 years [99]. A cross-sectional study from India found nearly 33% of older adults with diabetes at risk of cognitive impairment [100]. The Edinburgh Type 2 Diabetes Study showed that memory worsened progressively with the severity of hypoglycemia and diabetic retinopathy after adjustment for confounders. Similarly, stroke significantly accelerated the cognitive deterioration in a study over fifteen years. After adjusting for this macrovascular complication, the association between accelerated decline in cognition and diabetes was further reinforced compared to nondiabetics [101-103].

##### Pathophysiology

**Figure 2.**
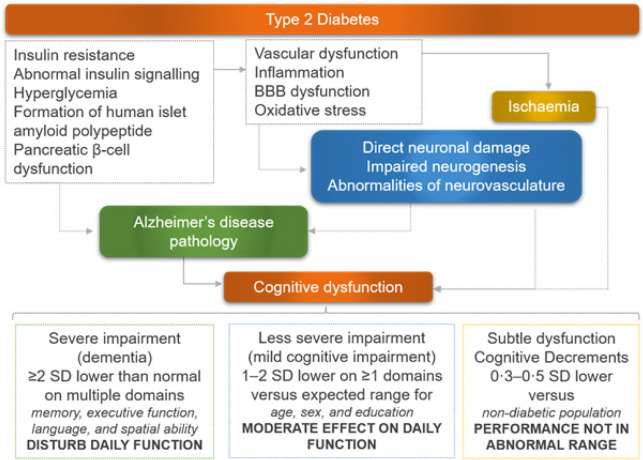
Type 2 diabetes and cognitive dysfunction.

Diabetes may increase the risk of neurodegeneration [96]. Chronic exposure to hyperglycemia can deteriorate cognitive function and other aspects of mental health (Figure 2). Reports have demonstrated that hyperglycemia is closely related to the development of cognitive impairment and dementia, suggesting that there may be a cause-effect relationship between hyperglycemia and dementia. Depression is a risk factor for the development of age-related cognitive dysfunction in people with diabetes, independent of vascular complications [104].

##### Investigations

Older diabetic patients with identified risk factors should be prioritized for a screening of cognitive impairment at the primary care level [100]. Annual screening for cognitive functions using simple appropriate tools (Mini-Mental State Examination Mini-Cog, and the Montreal Cognitive Assessment) should be conducted [105-110]. It is important to note that although robust data are missing currently, recognizing cognitive decline early will have a significant effect on diabetes management in these older adults. The cognitive assessment also should be conducted when glycemic control is impaired. Frequent episodes of hypoglycemia also should prompt cognitive assessment, because it could be due to the inability to identify hypoglycemic signals and consequently incapability of preventing it. Elderly with diabetes who are found to be positive on screening should be referred for prescribed cognitive/neuropsychological assessment.

##### Treatment

A cross-sectional analysis of the large PREDIMED-Plus study on the elderly suggested that once diabetes has been diagnosed, cognitive decline prevention strategies need to be implemented to improve treatment adherence and quality of life [111]. When cognitive decline is diagnosed, appropriate behavioural therapy should be initiated [112]. No disease-modifying therapy is currently available to cease or decelerate the processes that lead to dementia [113]. Only symptomatic (psychotropic) and standard (cholinesterase inhibitors (ChEIs) and a partial *N* -methyl-D-aspartate antagonist) AD therapies are currently available [114].

## 2 Geriatric Metabolic Syndromes and Diabetes

### 2.1 Hypoglycemia

#### Definition

Consistent with a Consensus Report from the ADA, AACE, and other Diabetes Associations three levels of hypoglycemia have been defined- level 1 or hypoglycemia alert (≤ 70 mg/dL); level 2 or clinically significant hypoglycemia (< 54 mg/dL) and level 3 or severe hypoglycemia, associated with severe cognitive impairment necessitating hospitalization for recovery (no specific glucose level).

#### Prevalence

There has been a steady rise in the occurrence of hypoglycemic episodes due to the increasing trends in achieving tighter glycemic control and prevalent co-morbidities. Hypoglycemia is the second most common adverse drug reaction. Insulin was the second most common medication associated with ED visits in older people ≥ 65 years, due to hypoglycemia [115,116]. The exact incidence of hypoglycemia is not known but it is higher in older than younger persons with diabetes. A one-year, prospective, observational study found significantly higher episodes in persons aged > 70 years than < 60 years old (12.8% vs. 9.0%, p < .01) and more persons in this group needed medical assistance (0.7% vs. 0.1%) [117]. The incidence is even higher in the community setting up to 41.9% over one year [118].

#### Causes

The counter-regulatory responses to hypoglycemia in persons with diabetes are defective [119]. The risk of hypoglycemia is increased in older adults due to several co-morbidities, including diminished hormonal regulation and counter-regulation, insufficient intake of water and/or food, decreased intestinal absorption, and cognitive impairment [120]. Polypharmacy, frequent in the elderly may increase the risk of severe hypoglycemia [121]. This patient population also demonstrates age-related changes (increased fat: muscle mass ratio, decreased renal function, decreased drug clearance) in pharmacokinetics and pharmacodynamics thus potentially increasing the risk of adverse events [121,122].

#### Presentation

In older adults, the counter-regulatory response depends on the frequency of hypoglycemic episodes they experience, with recurrent episodes reducing the glycemic level at which these mechanisms are activated [115]. Thus, the elderly with diabetes not only manifest fewer pronounced symptoms but also do so at progressively lower glucose levels compared to their younger counterparts. They tend to present with neuroglycopenic symptoms (i.e., dizziness, visual disturbances, increased agitation, and/or confusion) rather than classical adrenergic symptoms (i.e., palpitations, sweating, tremors), leading to loss of self-correction window [123]. Also, lack of cerebral glucose causes non-specific symptoms like nausea, falls, and unsteadiness. The neuroglycopenic symptoms are also non-specific because these may occur due to several other conditions, especially in the elderly [122,123]. Furthermore, such symptoms may occur even below the level associated with neuroglycopenia, a condition termed hypoglycemia unawareness. Thus, in older adults, hypoglycemia-associated autonomic failure can occur [115]. Therefore, due to the non-specific nature, multiple potential causes, and co-morbidities, recognizing hypoglycemia in the elderly is challenging.

#### Management

Periodic feedback to determine awareness of hypoglycemia, any associated symptoms, adherence to treatment, meal frequency, and unintentional repeat doses should be obtained from patients and their caregivers, (e.g., selected questions from the Diabetes Care Profile) [124,125 ]. Also, any future risk should be evaluated using validated risk calculators, and these older patients should be categorized based on the severity of anticipated hypoglycemia [126].

Consideration should be given to cognitive abilities and co-morbidities in older adults when determining the treatment strategy. The most obvious method is to select anti-diabetic regimens with a lower risk of hypoglycemia. Glycemic targets should be adjusted to avoid any risk of hypoglycemia, and at the same time, not enhance hyperglycemia [147].

### 2.2 Severe Hyperglycemia

Hyperglycemic hyperosmolar syndrome (HHS) is a particularly severe complication of unidentified or sub-optimally treated hyperglycemia in older adults. Dehydration, electrolyte abnormalities, urinary incontinence, dizziness, and falls should prompt appropriate glucovigilance to avoid HHS because it has a poorer prognosis in older than younger adults [127-129]. Providing a detailed approach to its management is beyond the scope of the current document.

### 2.3 Hypoalbuminemia

#### Definition

Hypoalbuminemia defined as serum albumin concentration < 1.5 g/dL [130].

#### Prevalence

In the data derived from the NHANES I population-based sample aged 55–74 years, diabetes was associated with an almost double risk of having a serum albumin concentration < 38 g/L. Also, reduced albumin is associated with an unfavourable metabolic profile [131].

#### Causes

Various studies demonstrate a progressive reduction of albumin serum concentration between 0.08 and 0.17 g/L/year, associated with aging [132]. Diabetes may be associated with impaired liver production of albumin because insulin is an important regulator of its synthesis [133]. Cross-sectional studies show that hypoalbuminemia is an independent risk factor for frailty in elderly with diabetes, suggesting relative malnutrition in these frail patients [133].

#### Presentation

Modest to very low serum albumin concentrations are associated with muscle wasting, disability, and higher morbidity and all-cause mortality in older persons [134]. Because albumin functions as a low-affinity, high-capacity carrier of several different endogenous and exogenous compounds acting as a depot and a carrier for these compounds, a drop in its serum levels may lead to increased free drug concentration in the plasma, more rapid hepatic metabolism, or both, which needs to be taken into consideration when treating diabetes and co-morbid diseases [135].

#### Management

Studies suggest serum albumin concentration of elderly people to be maintained at ≥ 4.0 g/dL to prevent frailty [136]. Such elderly with diabetes should be supported with sufficient high biological value protein and energy intake for anabolism.

### 2.4 Dyselectrolytemia

#### Prevalence

Among the various electrolyte abnormalities commonly found in the elderly, hypernatremia and hyponatremia (11%-18%) are the most frequent [137]. Both are associated with a high fatality rate. Based on the settings and the definition, hyponatremia ranges from 2.5% to as high as 50%. Electrolyte disturbances are frequent in persons with diabetes, particularly the elderly.

#### Causes

Dyselectrolytemia may occur due to altered dispersal of electrolytes corresponding to hyperglycemia-induced osmotic fluid shifts or total-body deficits owing to osmotic diuresis [138]. Elderly persons are at particular risk due to a decline in cognitive ability and may experience a syndrome of inappropriate antidiuretic hormone (SIADH) secretion [138]. Pharmacotherapies used for the treatment of diabetes also may result in electrolyte disturbances, specifically dysnatremia. Additionally, higher water intake in combination with a low salt diet adopted by many elderly patients and hypoproteinaemia due to diabetes and malnutrition may increase the risk of ‘Tea and toast’ hyponatremia [139].

#### Caution/Careful considerations

Because advancing age and diabetes (irrespective of hyperglycemia) per se increase the risk of dysnatremia, careful consideration of therapies that affect the electrolyte balance is necessary [140]. It is noteworthy that unlike TZDs and first-generation SU, second-generation SU rarely causes dysnatremia. SGLT-2i and GLP-1 agonists have not been found to cause electrolyte abnormalities [139,140]. Thiazide diuretics, and to a lesser, extent loop diuretics, induce hyponatremia [139]. Antidepressants associated with hyponatremia are predominantly attributed to SSRIs, SNRIs, and mirtazapine in persons ≥ 60 years of age [139]. Elderly persons treated with anti-epileptic drugs (AEDs) such as valproic acid, phenytoin, or topiramate have a higher risk of being hospitalized with hyponatremia. Other AEDs such as carbamazepine, oxcarbazepine, eslicarbazepine, lamotrigine, levetiracetam and gabapentin also induce hyponatremia [139].

### 2.5 Malnutrition

#### Prevalence

It has been suggested that the prevalence of malnutrition or its risk in elderly patients with diabetes is greater than 50% [141]. According to the Mini Nutritional Assessment (MNA), malnutrition is higher in the elderly with diabetes than in those without diabetes [141].

#### Effects on medication

Malnutrition is associated with functional impairment (decreases in activities of daily life, grip strength, physical performance of the lower limbs) and consequently poor quality of life, longer hospital stays, and increased rates of institution and mortality [141]. Malnutrition also plays a key role in developing frailty and sarcopenia. Muscle hypertrophy and atrophy may occur due to poor protein intake [141]. It can hinder normal brain function and encourage cognitive loss [141]. Thus, in elderly patients’ diabetes and its complications are compounded by malnutrition [142].

Besides the disease prolonged use of metformin causes vitamin B_12_ deficiency in 10%-30% of patients with diabetes. It may worsen cognitive performance in elderly patients [143]. However, vitamin B12 deficiency also has been reported in diabetic patients not taking metformin [144,145]. These factors should be specially considered in patients who develop paresthesia and neuropathy [146].

## 3 Goals for glycemic control in elderly

Considering the multiple co-morbidities and syndromic factors associated with advancing age, the usual glycemic targets recommended in younger adults are not justified in older adults with diabetes. In older adults with diabetes, the aim is to maintain an optimal quality of life, which in turn, depends on individual patient risk factors concurrently avoiding acute glycemic extremes.

Hypoglycemic events are a frequent occurrence in older adults due to diminished sympathetic counter-regulatory responses. Moreover, hypoglycemia predisposes this patient population to increased risk of diminished cognition, dementia, all-cause hospitalization, and all-cause mortality [147-149]. Hyperglycemia due to undertreatment increases the risk of dehydration, dizziness, falls, and long-term mortality [18,150].

Inadequacy of quality RCTs focussing on older diabetic adults impedes the establishment of suitable glycemic goals. Pivotal trials namely ACCORD, VADT, and ADVANCE trials in older adults have failed to demonstrate any additional benefit in cardiovascular outcomes with tight glycemic control as per AACE (A1c < 6% or < 6.5%), rather ACCORD was prematurely discontinued due to higher mortality (p = .04) and higher frequency of hypoglycemic events (p < .001) in the intensively treated group [151-153]. However, even an A1c level > 8% is associated with increased morbidity and mortality [154]. Based on this clinical evidence in older diabetic adults, rather than focussing on contentious glycemic targets, attuning/ personalizing the glycemic goals as a function of the patient’s duration of diabetes, life expectancy, functional status, concomitant disease conditions should be considered. Management should aim at a realistic and modest glycemic goal of A1c between 7% and 8% rather than tight control in old diabetic patients **(Table 3)** [155]. It is worth noting that although there are small differences in the A1c targets among guidelines, all of them recommend a personalized/individualized approach to diabetes management **(Table 4)**.

**Table 3.** Glycemic targets in elderly persons with Type 2 diabetes: Recommendations by various societies based on overall health status

Health and Functional Status		A1C		
	ADA	AGA	AACE	ACP
Most healthy older adults with intact cognitive and functional status	<7.5%	7-7.5%	≤6.5%	7-8%
Most frail older adults, with multiple co- morbidities and limited life expectancy	<8-8.5%	7.5-9%	>6.5%	No specific target but minimizing symptoms related to hyperglycemia

**Table 4.** Need for simplification/de-intensification of pharmacotherapy based on the overall health status of older patients with diabetes

Geriatric category	Factors affecting Functional capacity			Glycemic target	Calls for Simplification/deintensification
Group	Co-morbidities	Cognitive function	Functional status		
I Otherwise healthy, independent	few	+++	+++	Attain A1C	Severe or recurrent
					hypoglycemia on insulin Glycemic excursions
					Acute illness associated cognitive decline Polypharmacy
II Poor health, partiallydependent	multiple	++	+/-	Attain FPG and PPGgoals	Incapable of managing complex insulin regimen
					Absence of a caregiver or economic constraints
III Very poor health, completely dependent	multiple	-	-	Avoid hypoglycemia and hyperglycemia	To decrease the number of insulin injections On drugs with unclear benefits Inconsistent dietary habits
IVEnd of life, completely dependent	Limited life expectancy				On drugs that do not affect the quality of life
					To reduce discomfort associated with injections and reduce caregiver stress

‘The ABCDE experts agree that there is no ‘single best’ – the best varies for each patient, therefore instead of arguing on the glycemic goals, a tailored approach would be vital in maintaining an asymptomatic and quality life for the older adult with diabetes.’

### Prevention of falls

A major cause of morbidity and mortality in older adults with diabetes is falling; the risk in the elderly with diabetes is 17-fold higher compared to those without diabetes [156]. The three-year Longitudinal Ageing Study in Amsterdam found a 67% greater risk of recurrent falls (≥ 2 falls/6 months) in the elderly (> 65 years) with diabetes compared to those without which persisted after adjusting for other risk factors [157]. Overall, 18% of the cohort from the Study of Osteoporotic Fractures followed for more than seven years experienced recurrent falls. Compared to nondiabetics, diabetics treated with OHAs and insulin are at 68% and 178% higher risk of recurrent falls (> 1/year) [158]. These findings for insulin were substantiated again by the five-year Kaiser Permanente Registry in the elderly (> 60 years) [159]. Diabetes is also known to increase the risk of psoriatic, gout, and rheumatoid arthritis, causing movement-limiting pain and mobility weakness. Persons with diabetes are also at twice the risk for osteoarthritis compared to the general population.

It is essential to screen and modify the risk factors for falls in elderly persons with diabetes and formulate interventions tailored to address the detected issue (balance, walking dysfunction, reactions, muscle weakness, pain) **(Table 5)** [156,160,161].

**Table 5.** Falls/fractures: causes and Interventions for prevention [150,160,161]

Causes/Risk factors for Falls/Fractures	Multifactorial Preventive Interventions
Hypoglycemia, hypotension, postural hypotension	• Reduce the intensity of glycemic control • Adjust antihypertensive and psychoactive medication • Treat postural hypotension
Diabetes-related loss of strength, arthritis, pain	• Structured exercise • Balance training (yoga, tai chi) • Aerobic exercise • Resistance training • Co-ordination training
Loss of sensor y perception, balance secondar y to peripheral neuropathy, ataxia	• Agents to relieve neuropathy signs and symptoms • Medical nutrition therapy, vitamin B12, vitamin D supplementation
Secondar y hyperparathyroidism and renal osteodystrophy related to DPN	• Treat underlying pathology
Polypharmacy	• Reduce polypharmacy to improve cognitive function
Cognitive impairment	• Cognitive training/therapy
Dyselectrolytemia	• Correction of electrolyte levels
Psoriatic, gout, and rheumatoid arthritis pain	• Immune therapy
Reduced visual acuity due to cataracts/ macular degeneration/ glaucoma	• Treat accordingly
Arrhythmia	• Treat accordingly
Foot problems (plantar pressures, ulcer, bony prominences) causing discomfort restricting movement	• Use orthotic devices (insoles, rocker-bottom shoes) • Orthoses compensate for age-related changes (improve ground contact, house boney eminences, and alleviate skeletal imbalances) • Reduce pain and improve the ambulatory capability
Footwear	• Encourage patients to wear low-heeled shoes

## 4 Management

### Non-pharmacological Interventions

#### Lifestyle management

In the elderly with diabetes, lifestyle management should be tailor-made to their frailty status. Optimal nutrition with sufficient protein intake along with a structured exercise program is aimed at decreasing sedentary intervals and increasing mobility in frail elderly persons with diabetes. In the elderly who are not frail, but are overweight or obese, given its multiple benefits, an intervention aimed at weight loss is recommended [162].

#### Diet and Nutrition

Nutritional status should be assessed for older patients with diabetes for the preclusion of cognitive impairment, frailty, and mortality is BMI ≥ 30 kg/ m2, weight gain (> 10%), metabolic syndrome (< 75 years of age), sarcopenic obesity, BMI < 18.5 kg/m^2^, inadvertent weight loss, and malnutrition. A dietitian’s referral should be advised in this regard because they provide advice for maintaining nutritional status and medical nutrition therapy, when necessary, bearing in mind the patient’s personal preferences (age-related deterioration in taste, dental problems, co-morbidities, dietary restrictions, poor gastrointestinal function), food availability, and individual goals which improve QoL [162,163] (**Table 6)**.

**Table 6.** NPI in elderly persons with diabetes: nutrition essentials [162,165]

**Protein intake**	Attenuates the decline in muscle mass and insulin sensitivity that occurs with increasing age and diabetes.
	Helps in an increase in protein anabolism, ‘high-quality’ weight loss (fat loss and muscle preservation/gain), enhanced glycemic control, daily appetite control, and satiety.
	Protein intake should be 1.0-1.2 g/kg/day in older adults and 1.2–1.5 g/kg/day in those who are at risk of malnutrition.
**Dairy foods**	Increased consumption may be beneficial for older adults with diabetes due to the combined effect of dairy on both insulin sensitivity and lean body mass.
**Diet rich in fruits and vegetables as well as exogenous antioxidant vitamins (such as vitamins E and C and Carotenoids) and minerals**	Restores the skeletal muscle redox homeostasis and prevents oxidative stress, thereby contributing to muscle maintenance.

#### Exercise and Physical activity

Frailty prevalence is ~10% in the population over 60 years old and increases to > 25% in those ages ≥ 80 years of age [164]. A cross-sectional study showed that compared to Europeans, South Asians (~50 years of age) need to undertake ~230 minutes of moderate-intensity physical activity every week [165].

In older adults, apart from improved glycemic benefit, exercise also improves body composition and arthritic pain, reduces falls and depression, increases strength and balance, enhances the quality of life, and improves survival [166-168]. It is prudent to get the patient to start an exercise or physical activity program gradually and encourage them to remain consistent, with recommended activities based on functional status **(Table 7)** [169-171].

**Table 7.** NPI for elderly persons with diabetes: physical activity [162,169,170]

Elderly with diabetes	Intervention	Goal
Frail- mobility, and gait issues at high risk of fall/fractures Cognitive impairment, vision impairment	Physical therapists and occupational therapists Weight training and aerobic exercise	Improve balance and muscle strength Improve mobility to enhance functional capacity
Non-frail, independent	Aerobic exercise 30 minutes for 5 days/ week	Maintain mobility and cardiovascular health
High risk of CVD	Walking or aerobic exercises (routine ECG/stress test)	To reduce the risk of coronary heart disease and cerebrovascular disease

#### Stress Management

Chronic diseases like diabetes and associated socioeconomic factors can be major causes of stress among the elderly. Chronic psychological stress may increase inflammatory and platelet aggregation responses and result in poor diabetes control due to activation of the hypothalamic-pituitary-adrenal axis [172]. If such stressors are not managed, the elderly are at risk of developing mental health issues like anxiety and depression. Considering that psychosocial impact affects morbidity and mortality in persons with diabetes, psychosocial facets should be included at all levels of diabetes management. Thus, elderly persons with diabetes require access to stress management along with treatment of metabolic abnormalities to improve treatment adherence to achieve good glycemic control, reduce the risk of complications and improve QoL [173]. Psychosocial stress management could include Cognitive behavioral therapy, motivational therapy, problem-solving therapy, coping skills training, and family behavior therapy **(Table 8)** [174-178,180,189].

**Table 8.** NPI for elderly persons with diabetes: stress management methods

Method	Description
CBT	• Recognition of irrational or rational thoughts which contribute to the stress. • Using CBT these dysfunctional thoughts on diabetes and other life issues should be minimized. • The elderly should be educated to practice questioning themselves in stressful situations • [Questions such as ‘Is my handling of this concern appropriate or helpful? What would be helpful, appropriate handling (thought, behaviour)? Can the problem be pushed aside without worrying?]
Problem-solving strategies	• It is important to identify patients’ exaggerated fears or worries as well as extreme callousness regarding disease and treatment. These issues should be addressed using problem-solving strategies.
Motivational interviewing	• Motivational interviewing self-management program leads to significant improvement was found for self-efficacy, self-care behaviour, glycemic control, and quality of life (daily life satisfaction, influence of disease) • Assessment of perception of self-management level, hurdles, and coping strategies for self-management and goal setting should be performed. • Use of verbal persuasion and appreciation for sustaining self-care. Reassess barriers and offer more coping strategies if goals are not met.
Family behaviour therapy	• Involving family members and caregivers in interventions for the elderly population may be vital for providing them with social support related to their diabetes management. • Spouse participation in diabetes education program for elderly diabetic patients showed greater improvements in knowledge, metabolic control, and stress level than those who participated alone. • Such programs involving family social support training have also helped older patients to maintain dietary restrictions.
Social Interaction	• Communication between social groups for the elderly at sports centres, local parks, religious centres may improve selfmanagement skills and the elderly may also find motivation from patients of the same age-group

#### Sleep Hygiene

Diabetes can cause direct sleep disturbances due to nocturia, polyuria, diabetic neuropathy and neuropathy pain, and other several chronic illnesses that can impair sleep and quality of life [181]. It is important to address their sleep issues and the impaired quality of life due to inadequate and fragmented sleep, as it may be severely affecting their glycemic control and quality of life. Therefore, sleep education is an essential aspect of diabetes management in the elderly **(Table 9)** [182-184].

**Table 9.** NPI for elderly persons with diabetes: methods to improve sleep hygiene [182-184]

• The patient should be educated to maintain a pre-sleep routine, the same sleeping and waking hours (7-8 hours) • Advice getting sufficient sunlight during the day • Suggest using a fan or white noise machine to block disturbances • Advice sleep in a quiet dark room and use of comfortable bed and clothes • If unable to fall asleep, suggest reading, listening to soothing music, and trying to sleep again • Advice avoiding day-time napping • Advice avoiding CNS stimulating substances (caffeine, alcohol, tobacco) and activities (screens- television, mobiles, computers) 90 minutes before bedtime • Advice avoiding spicy, heavy dinners

#### Environmental Modulation

Aging with individuality is vital for the elderly which includes living independently in one’s home and choosing on spending one’s time [185,186]. These aspects are influenced by an interaction between health and the environment. Physical environmental barriers can generate hazards in the home and community. Elderly individuals with chronic conditions and functional limitations are not only at significant risk for adverse health events (such as falls) and injuries but also face difficulties in performing ADLs. Moreover, these barriers attenuate the effectiveness of caregivers, assistive technologies, and health care devices sometimes necessitating early institutionalization **(Table 10)** [187,188].

**Table 10.** Environmental modulation at homes and communities for elderly care

• Availability of lifts • Steps should be in good repair, with handrails on both sides and with contrasting nosing • Walkways and floors (homes/ bathrooms) should have smooth, slipresistant surfaces, use non-skid bathmats • Walkways may be replaced with a ramp, sloping walkway, or mechanical lift • Doorways should be widened, sufficient space should be provided to maneuver • An automatic opening system should be installed to eliminate the twisting and turning of doorknobs. • Adequate lighting operated by motion detectors or timers at all walkways and doors to help maintain independence • Rearrange furniture • Hang windchimes/Install door alarms/ alerts (cognitive disability- reduce risk of wandering away) • Rugs should be anchored to prevent slipping • Minimize clutter in the house • Remove loose wires	• Sufficient space should be available at the toilet, bathtub, shower, and sink for mobility aids and caregiver assistance. • Reduce the distance an individual must raise and lower himself or herself (e.g., raising the height of the toilet) or the need to lift one’s legs over the side of the tub (walkin tub) or the shower curb (curbless shower) to enhance safety • Facilitate transfers by adding supports (such as grab bars, safety frame, or floor-to-ceiling pole) or using a fixture with integral supports and increasing the visibility (contrasting color of the toilet or toilet seat from walls) of all fixtures.

*‘Experts suggest that given the less consultation time (~3.5 minutes) and large patient pool, physicians should recruit dietitians and patient care educators for patient benefit since lifestyle changes form the cornerstone of diabetes management.’*

#### Pharmacological

The pharmacotherapy for the elderly should be directed at achieving good glucose control, without causing hypoglycemia, and preventing symptomatic hyperglycemia. Intensive glycemic control should be avoided and so should polypharmacy. Polypharmacy increases the risk of drug-drug and drug-disease interactions, particularly in elderly persons with diabetes with sensory and cognitive deficits due to age [189]. Such deficits may impede the prompt conveying of initial symptoms of adverse drug events. Therefore, pharmacotherapy for the elderly person with diabetes should be chosen based on individuals’ functional capabilities. These include but are not limited to visual, motor, or cognitive abilities, pre-existing comorbidities, and risk factors **(Table 11** and **Table 12)**.

**Table 11.** Pharmacological management of elderly persons with type 2 diabetes mellitus

Drug Class	Contraindications	Caveats	Checkpoints (monitoring)	Dose adjustnent		Strengths/Advanfijges
Biguanide Metformin [190-192]	• Acute metabolic acidosis (such as lactic acidosis, diabetic ketoacidosis) • Diabetic procoma • Severe renal failure (GFR <30 mL/min) • Acute conditions altering renal function (dehydration, severe infection, shock)	Risk of lactic acidosis (Discontinue in older adults with risk factors such as age > 80 years, gastrointestinal complaints during the last year, and/ or GFR ≤60 ml/ min)[193] Risk of gastrointestinal adverse effects weight loss, diarrhea, and loss/ reduced appetite -concerning effects in frail older adults due to poor nutritional reserves Risk of vitamin B12 deficiency	Regular assessment of renal function is necessary (before initiation and every 3-6 months thereafter) Monitor vitamin B12 levels	Due to the potential for decreased renal function, the metformin dosage should be adjusted based on renal function.		• Low risk of hypoglycemia • Simple dosing regimen • Pleiotropic effects • Improves physical function • Limits ceramides in skeletal muscles- Helpful in sarcopenic obesity, Improves muscle strength, mobility, endurance • Weight loss-small but significant (may become a cause of worry in some elderly) • Enhanced benefits against the aging process • Improves endothelial function, reduces inflammation, and improves angiogenesis • Cardioprotective- stimulates ischemia-induced revascularization • NeuroprotectivoReduces neuronal injury and improves oxygen/ glucose deprivation • Reduces MCI and risk of dementia • Improves depressive symptoms
	• Conditions causing tissue hypoxia (acute disease, or worsening of chronic disease- decompensated heart failure, respiratory failure, recent myocardial infarction, shock) • Hepatic insufficiency, acute alcohol intoxication, alcoholism			eGFR (mL/min) 60-89 45-59 30-44	Dose (mg) 3000 2000 1000	
Traditional Sul- phonylureas Glibenclamide AGS Beers criteria [194]	Elderly: not recommended above 65 years due to very high risk of hypoglycemia					
Modern Sulfo- nylureas [195] Gliclazide Glimepiride^1^	• Diabetic coma • Ketoacidosis • Severe renal or hepatic function disorders	Risk of Hypoglyce- mia^2^ [196] (Irregular meals, undernutrition, frailty impaired renal function, serious hepatic dysfunction) Multiple drug inter- actions^3^	Regular monitoring of glucose levels in blood and urine Periodic A1C Regular hepatic and hematological monitoring (especially leucocytes and thrombocytes)	Change in weight or lifestyle, change the dosage		• Simple dosing regimen • Adequate glycemic control
**Meglitinides** Repaglinide	• Diabetic ketoacidosis, with or without coma • Severe hepatic function disorder • Concomitant use of gemfibrozil	Increased risk of hypoglycemia in combination with other antidiabetic agents Increased incidence of the acute coronary syndrome (e.g. myocardial infarction) Multiple drug interactions Increased plasma levels in patients with hepatic insuffi- ciency, severe renal impairment, and in the elderly	If concomitant use of interacting drugs is necessary, careful monitoring of blood glucose and close clinical monitoring should be performed	Dose adjustments due to drug interaction		• Rapid onset • Reduced renal function or those experiencing troublesome hypoglycaemia with sulfonylureas, use Repaglinide
Thiazolidinediones (Pioglitazone) ^a^	• cardiac failure or history of cardiac failure (NYHA stages I - IV) • hepatic impairment • diabetic ketoacidosis • current bladder cancer or a history of bladder cancer • un-investigated macroscopic haematuria	Combination use with insulin cause fluid retention and should be considered with caution because of the increased risk of serious heart failure. Increased risk of bone fractures (particularly in elderly women)- avoid in persons with pre-existing osteoporosis Increased risk of bladder cancer Consider the balance of benefits and risks carefully both before and during treatment due to age-related risks Risk of macular edema May reduce the risk of dementia [197]	Watch for signs and symptoms of heart failure, weight gain, or edema; particularly those with reduced cardiac reserve. Monitor older adults with low bone mass (osteopenia), for changes in bone density	No dosage adjustment is necessary due to age or impaired renal function (CrCl> 4 ml/ min)		• Improved lipid profile • Decreased risk of MI, NASH • Protects against dementia
**AGIs** Acarbose, Voglibose, Miglitol	• Inflammatory bowel disease, colonic ulceration, partial intestinal obstruction, or at risk of intestinal obstruction. • Chronic intestinal diseases associated with marked disorders of digestion or absorption • Patients vulnerable to increased gas formation in the intestine (larger hernias) • hepatic impairment. • Severe renal impairment CrCl <25 ml/ min/1.73m^2^	Gastrointestinal discomfort Frequent dosing (thrice daily) Avoid sucrose-containing foods	Liver enzyme monitoring for the first 6 to 12 months of treatment Withdraw therapy if transaminases are elevated and monitor weekly until normalization	• No dosage adjustment is necessary due to age • Adjust dosage since it potentiates the hypoglycaemic effects of insulin, metformin, and SU drugs		Rapid onset Antihyperglycemic effect (No hypoglycemia)
**DPP-4 inhibitors** Sitagliptin, vildagliptin, saxagliptin, linagliptin, teneligliptin, evogliptin	No specific contraindications (Only hypersensitivity)	Not to be used in hepatic impairment Risk of pancreatitis Risk of hypoglycaemia with SU	Monitored liver enzymes during treatment at three-month intervals during the first year and periodically If increased monitor liver functions until normal Withdraw if increase in AST or ALT of 3x ULN, do not reinitiate Monitor for skin disorders, such as blistering or ulceration	• No dosage adjustment due to age • No dosage adjustment due to mild renal impairment • Reduce dose by 50% in moderate to severe renal impairment • Adjust SU dose in combination with DPP-4i		• Low risk of hypoglycaemia (a glucose-independent mechanism) • Few gastrointestinal side-effects • Weight neutral • Low potential for drug interactions • No significant pharmacokinetic effects of age in elderly • Efficacy similar to younger persons with diabetes • Better glycemic control and fewer adverse events compared to metformin (Vildagliptin) • It may have cognitive benefits (Sitagliptin)
**Injectable** **GLP-1 RA** Liraglutide **Dulaglutide** **Semaglutide**	No specific contraindications (Only hypersensitivity) Not recommended for use in patients with severe hepatic impairment	Risk of pancreatitis Caution in patients with thyroid disease Risk of hypoglycaemia with SU The potential risk of dehydration associated with GI side effects (nausea, vomiting, diarrhea)-the risk of weight loss in frail elderly Avoid fluid depletion.		• No dose adjustment is required for patients with mild, moderate, or severe renal impairment, mild or moderate hepatic impairment		• Lower risk of hypoglycemia • Self-monitoring of blood glucose is not needed to adjust the dose • Weight loss • No differences in the efficacy and safety profile between elderly and younger patients • Reduction in major adverse cardiovascular events (MACE) in patients with established CVD and potentially for those at high risk for CVD [198]
**Oral GLP- 1RA** **Semaglutide**	No specific contraindications (Only hypersensitivity) Not recommended for use in patients with severe hepatic impairment	Risk of pancreatitis Caution in patients with thyroid disease Risk of hypoglycaemia with SU The potential risk of dehydration associated with GI side effects (nausea, vomiting, diarrhea)- the risk of weight loss in frail elderly Avoid fluid depletion.		• No dose adjustment is required for patients with mild, moderate, or severe renal impairment, mild or moderate hepatic impairment		• Lower risk of hypoglycemia • Self-monitoring of blood glucose is not needed to adjust the dose • Weight loss • No differences in the efficacy and safety profile between elderly and younger patients • Non-inferior to placebo in reducing MACE in patients with established CVD and potentially for those at high risk for CVD [198,199]
**SGLT-2 inhibitors** Canagliflozin, Dapagliflozin, Empagliflozin, Remogliflozin	No specific contraindications (Only hypersensitivity)	• Limited experience in patients aged ≥85 years • Extensive evidence in age group lacking • In patients ≥ 75 years, consider the increased risk for volume depletion • Special attention to volume intake with concomitant volume-depleting agents (e.g. diuretics, ACE inhibitors). • May increase the risk of dehydration and hypotension in combination with diuretics • Risk of urinary tract infections, and worsening urinary incontinence • Increased risk of lower limb amputation • Use with caution in patients at high risk of fracture (low BMD or history of falls) or amputation (vascular disease, diabetic foot ulcer, or previous amputation) • Increased risk of hypoglycemia with SU/ insulin • Risk of necrotizing fasciitis of the perineum, (Fournier’s gangrene) • Increase in serum lipids • Weight loss- Caution must be used when prescribingSGLT2i in older patients with nutritional risks	• Treatment should be interrupted and ketones monitored in patients who arehospitalized for major surgical procedures or acute serious medical illnesses • Assess renal function before initiation and periodically during treatment (> yearly) • Before initiation of any concomitant medicinal product that may have a negative impact on renal function • Monitor volume status [physical examination, blood pressure measurements, laboratory tests (haematocrit), and electrolytes] in case of conditions that may lead to fluid loss	No dose adjustment is recommended based on age. Dose adjustment as per eGFR (mL/min		• Lower risk of hypoglycemia • Weight loss • OD dosing (simple dosing) • Mild diuretics, slight benefit in fluid and blood pressure reduction • CV benefit (Empagliflozin, Canagliflozin) • Reduce diabetic nephropathy progression (Canagli-flozin, Dapagliflozin)
				Type 2 diabetes		
				≥60	Initiate with lower strength Can be increased to higher strength	
				45 to <60	Initiate and Continue with lower strength	
				Type 2 diabetes with heart failure		
				≥20	Initiate with lower strength	
				<20	Not recommended	
				Carefully monitor for genital infections		
**Human Insulin**	No specific contraindications (Only hypersensitivity)	Higher risk of hypoglycemia Hypoglycemia unawareness (autonomic neuropathy, long history of diabetes, suffering from a psychiatric illness) In combination with pioglitazone, increased risk of heart failure Medication errors Multiple drug interactions	• Close monitoring for hypoglycemia risk factors • Monitor urinary ketones	Dose adjustments are necessary based on hypoglycaemia risk, urinary ketones, renal function, hepatic impairment, and concomitant drug therapy		• Rapid onset
**Insulin analogs**	No specific contraindications (Only hypersensitivity)	Preferred in patients with • hypoglycemia unawareness • frequent hypoglycemia • brittle diabetes	• Close monitoring for hypoglycemia risk factors • Monitor urinary ketones	Dose adjustments are necessary based on hypoglycaemia risk, urinary ketones, renal function, hepatic impairment, and concomitant drug therapy		• Rapid onset

(Endnotes)

^1^ Added to AGS list Updated AGS Beers Criteria® for Potentially Inappropriate Medication Use in Older Adults

^2^ Hypoglycemia may trigger serious events like myocardial infarction or stroke, and can lead to permanent neurological damage and even death

^3^ Reduce SU efficacy- Diuretics Diphenylhydantoin Glucocorticoids Lithium Rifampin Isoniazid Nicotinic acid; Increase hypoglycemic effect Sulfonamides Salicylates Clofibrate Dicumarol MAO inhibitors NSAIDs β-Adrenergic-blocking agents Alcohol

**Table 12.** Clinical evidence that forms the basis for pharmacotherapy recommendations in elderly persons with type 2 diabetes

Drug		SRMA (year/N/age)	Result	Conclusion/Recommendation	Report of the functional level and cognitive status of the participants	
Biguanide (Metformin)		Schlender (2017/ 230,229 ≥65 years) [193]	• Metformin was not significantly different than usual care (non-specific active treatment) for serious adverse events • A combination of insulin glargine plus glimepiride and metformin demonstrated significantly fewer hypoglycaemic events compared to premixed insulin. • Significantly fewer deaths in participants taking metformin compared to participants taking either no insulin sensitizer, no antidiabetic drugs [38], or no metformin. • No significant difference in mortality for patients >80 years, or for those patients with GFR. ≤60 • Re-admission for heart failure was significantly lower with metformin • No increased risk of lactic acidosis	• Lowers mortality risk versus no insulin sensitizer, no OHA, no metformin • Reduces risk of adverse events such as hypoglycemia and nonfatal cardiovascular events, than sulfonylureas. • Data particularly in very old (≥ 80 years), functionally and cognitively impaired older people lacking. • Consider discontinuation in those with limited life expectancy or functional impairment - very old people - renal insufficiency (GFR ≤60 ml/min) - gastrointestinal complaints during the previous year	No	
Sulphonylurea		Graal (1999/ pharmacoepidemiological study 14 000; Medicaid participants 20 000 >65 years; 21 outpatients mean age 70 yr) [196]	Glycaemic control was equivalent with glibenclamide and gliclazide but the incidence of hypoglycaemic (symptomatic, serious) reactions was significantly greater (twice) with glibenclamide [200-203]	Glibenclamide contraindicated Risk factors for severe hypoglycaemia [202] - recent hospital admission - advanced age (>80 years) - Extensive use of comedication Decreasing endogenous (β-cell function may limit the therapeutic efficacy of sulfonylureas [204]	No	
TZDs		Graham (2010/227,571 ≥65 years) [205] Lipscombe (2007/ (N=159026) Masoudi (2005/16 417)	Use of rosiglitazone was associated with an increased risk of stroke, heart failure, and all-cause mortality and an increased risk of the composite of AMI, stroke, heart failure, or all-cause mortality in patients ≥65 years. Higher risk of readmission for heart failure with TZD treatment and lower risk with metformin treatment	Not be used in patients with heart failure NYHA class III orIV. Treatment decisions must remain individualized, with clinicians weighing the potential benefits and harms of TZD treatment, especially among high-risk elderly populations	No	
		Lee (2011/3,752 ambulatory men aged ≥65 years) [206]	Lose less lean muscle mass	Beneficial for older adults, frail elderly patients, but the downside is increased risk of fractures, probable macular edema, heart failure, and fluid retention		
		Ye (2016/544,093 participants, age not stated) [197]	The incidence rate of dementia was reduced with thiazolidinediones, with a marginal trend toward significance	Might protect against incident dementia	Yes	
		Schwartz (2006/69/7079 years) [207]	BMD loss only in women, proportional to the duration of use Use for continuously 5 years, average BMD loss 3% more than expected.	Patients with diabetes already have an increased risk of fracture. TZDs associated with bone loss and increased risk of bone fractures, in elderly		
		Solomon (2009/20,964 Medicare beneficiaries, ≥ 65 years) [210]	TZDs may be associated with an increased risk of fractures compared with oral sulfonylureas and metformin.	women [208,209]. Not recommended for use in older adults with preexisting osteoporosis, if prescribed BMD needs to be closely monitored		
DPP4 inhibitors	Schott (2017/?/≥65 years) [211] 30 studies: 1 meto-analysis, 17 intervention studies, and 12 observational studies. 16 studies were focused on older adults and 14 studies reported subgroup analyses		Similar or better safety than placebo and other antidiabetic drugs Safety data are mainly based on shortterm outcomes like hypoglycemia in studies with A1C control levels recommended for younger people.	Lower risk of hypoglycemia, but data show conflicting findings for long-term benefits Reconsider the use in older adults with A1C <8.5% because of scarce data on clinically relevant benefits	Comorbidities were reported in 26 studies and frailty or functional status in a single study
	Yu (2020/300, 73.7 ± 9.1 years)		More than 12 months of use leads to a significant decrease in liver transaminase- decreased incidence of fatty liver in elderly diabetic patients No significant change in serum creatinine level and creatinine clearance Significant decrease in body weight and BMI No hypoglycemic reaction and gastrointestinal discomfort		
	Viljoen (2013/431/74 years)		Significantly less hypoglycemia in DPP-4Í vs non-DPP-4i group		
Sitogliptin	Isik (2017/253/74-76 years) [212]		Significant increase in the MMSE scores with sitagliptin vs metformin alone in pts with and without AD	Improvement of cognitive function in elderly diabetic patients with and without AD	Yes
Vildagliptin	Bulut (2020/130/75 years)		Improvement in MMSE scores (copying subdomain) at end of 6 months. Reduces A1C and body weight	Improves cognitive function and metabolic control	Yes
Vildagliptin	Pratley (/374/>65 years) [213]		A meta-analysis found a difference in effects on A1C, fasting plasma glucose, and body weight between younger and older patients treated with vildagliptin. Older patients showed an even lower incidence of adverse events than younger patients; Confirmed hypoglycemia occurred in lesser older vildagliptin population vs younger		Yes
	Blonde (2009/533/≥65 years) GALIANT Trial [214]		vildagliptin, vs placebo or metformin as monotherapy in patients aged > 65 years.	Vildagliptin was shown to be effective, safe, and well-tolerated in a wide sample of older patients, which was characterized by an elevated prevalence of mild renal insufficiency, multiple comorbidities, and polypharmacy Vildagliptin has similar efficacy to TZDs as an add-on to metformin over 3 months regardless of race, age, or BMI	Yes
	Schweizer (2011/301/77 years) [215]		Pooled analysis showed that in the very old patient population with comorbidities and polypharmacy, vildagliptin led to a clinically relevant A1C reduction either as monotherapy or add-on Has a similar safety profile in younger and older patients.		
	Ligueros-Sylan (2010) Schweizer (2011)		Meta-analysis showed that vildagliptin was not associated with increased risk of hepatic events or hepatic enzyme elevations indicative of drug-induced liver injury, pancreatitis, infections, or skin-related toxicity [216] Renally-impaired vildagliptin-treated very old patients reported a lower incidence of overall AEs than comparators [215].		
	Lukashevich (2011)		Largest (n = 515) DPP-4 inhibitor study in moderate to severe renally-impaired diabetic patients, characterized by longstanding diabetes (≥ 16 years) and advanced mean age (≥ 66 years). Vildagliptin treated patients experienced clinically relevant A1C reduction (approximately by -0.8%) with a similar incidence of overall AEs (including hypoglycemic episodes) to placebo [217]			
Vildagliptin Sitogliptin	Scheen 2010		Very unlikely to be responsible for drug-drug interactions with commonly prescribed medications in everyday clinical practice, as vildagliptin is not a cytochrome P450 (CYP450) substrate nor does it inhibit or induce CW450 engine [218]			
Sitogliptin	Barzilai 2010		206 patients aged > 65 years (mean age: 72 years), compared monotherapy with placebo, observing a significant reduction of A1C without hypoglycemic risk [219]			
	Herman 2010		A pooled safety analysis from 19 studies did not show any difference in efficacy or safety across the different ages [220]			
Saxagliptin	Doucet 2011		efficacy and safety in persons aged > 65 years (mean age: 69 years) appeared to be similar to those observed in younger adults [221]		No	
Saxagliptin	GENERATION Study Schernthaner (2015/720/72 years)		Superior to glimepiride for patients aged <75 years and inferior for patients aged ≥75 years Incidence of confirmed/severe hypoglycemia lower with saxagliptin vs glimepiride		No	
GLPIRA	Karragiannis (2021/93,502/≥65 years) [198]. LEADER, SUSTAIN 6 REWIND		GLP-1 RAs reduced MACE, cardiovascular death, stroke, and myocardial infarction [222]	Beneficial in those with MACE (Liraglutide, Injectoble Semaglutide, Dulaglutide)		
	Sun and colleagues (meto- analysis 2015) [223].		Modest decreases in LDL-C, triglycerides, and total cholesterol Significantly reduce SBP and DBP	Multiple pleiotropic effects that can help reduce polypharmacy in older diabetes patients with co-occurring chronic diseases (e.g. fatty liver disease and hypertension) and syndromes (such as sleep apnea and Parkinson's disease).		
	Multiple clinical trials and meta-analyses [189].		Improved hepatic function in NAFLD as measured by transaminase levels, biopsy, and images			
			Prevented the expected decline of cerebral metabolic rate for glucose uptake in the parietal-temporal frontal and posterior cingulate cortices.	Neuroprotective, and even regenerative, properties may be utilized in patients with AD or PD		
	Cochrane review 2017 [224].		No relationship between the use of diabetes medications and cognitive decline during a follow-up period of 40- 60 months			
	McIntyre (meta-analysis 2013) [225]. Billings and colleagues (2018)		Improvement in mood and cognition with GLP-1 RAs, Weekly GLP1RA had greater health- related quality of life (i.e. improved treatment satisfaction and willingness to continue treatment) vs. daily Weekly semaglutide - greatest treatment satisfaction.	The cognitive effects of GLP-1 RAs are especially important in patients with diabetes, given the high prevalence of cognitive impairment and antipsychotic related metabolic syndrome in this population		
SGLT-2Í	Karragiannis (2021/93,502/≥65 years) CANVAS program, DECLARE-TIMI 58, EMPA-REG OUTCOME, CREDENCE, ^RTIS CV (Mean age >63 years)		SGLT2 inhibitors reduced MACE but the effect on components is neutral Reduced heart failure hospitalization, CV mortality, and the composite renal endpoint Meta-analyses for patients ≥75 years yielded similar results. Weight loss (visceral fat loss) [226] SBP reduction [227]	Beneficial in those with MACE, HF, and renal ailments Useful in sarcopenic obesity Assess Functional status and renal function [228] Initiate with caution, especially in the adjustment of concomitant therapies, such as insulin and antihypertensive drugs, particularly loop diuretics [228]	Yes	
	Pooled analysis of Dapagliflozin studies [229]. (2016/1449/>65 years)		Occurrence of genital and urinary infection events were not increased with more advanced age	Guidance to improve personal hygiene after urination, when starting treatment Higher number of genital adverse events is not intensified in the older population. Standard treatment with no need to interrupt SGLT2Í. Re-evaluate use in case of repetitive events Risks versus benefits must be analyzed and use must be carefully monitored		
	DECLARE-TIMI 58 [230]		No greater rate of occurrence of Fournier syndrome versus placebo Proposed cause: diabetes-related compromised immunity, not SGLT2-I per se			
	Pooled analysis- Canagliflozin [231] DECLARE-TIMI 58 [232] EMPA-REG OUTCOME [233]		The occurrence of hypoglycemic events was not increased with more advanced age			

MMSE-Mini-Mental State Examination; MACE- major adverse cardiovascular events.

#### Therapeutic parsimony

Geriatric physiology and psychology are different from those of younger and healthy adults [234]. Due to age-related changes in pharmacokinetics and pharmacodynamics, elderly persons respond to lower doses of several drugs [235]. Furthermore, they also occasionally forget to take their medicines at the right dose and at the right time. Unfortunately, however, polypharmacy becomes more common with advancing age. This is attributed, at least in part, to the presence of multiple co-morbidities and complications in this population.

The law of therapeutic parsimony provides us with a framework in which to plan therapy for geriatric persons living with diabetes. It states the following: *‘minimal therapeutic interventions should be used, in place of multiple ones, as long as this can achieve equivalent therapeutic outcomes’* [236]. Being cognizant of this law, an attempt should be made to minimize the number of tablets/capsules or pill burden, and the frequency of their administration. A few means of achieving this end include prescribing rational fixed-dose combinations, which in turn, reduce the dose of individual agents. Agents with proven pleiotropic effects also could be preferred, provided adequate efficacy is assured. It should be suggested to the patient that all medications can be taken at the same time, perhaps five minutes before a meal or immediately after a meal. It also should be ensured that while prescribing brand names, strips or tablets that are similar in appearance are not prescribed. It is common for the patient to get confused between two strengths of the same medication; e.g., 1 mg and 2 mg of glimepiride tablets due to similarity in packaging.

#### Glycemic Monitoring

Optimal glycemic control in elderly persons with diabetes can be achieved through certain lifestyle adaptations, adjustment, and individualization of the therapy, which invariably depends on careful self-monitoring and control. Blood glucose monitoring can be done by different methods **(Table 13)**.

**Table 13.** Methods of monitoring glycemic control in elderly persons with diabetes: benefits and limitations [29,237,238]

Method	Need	Benefits (in elderly*)	Limitations (for elderly*)
Glycated hemoglobin (A1C)	Laboratory-based test	• Represents the average the exposure • Represents risk of complications	• No information on daily glycaemic values • No information on daily glycaemic variability • Unable to differentiate between pre-prandial and postprandial glycemia, possible hypo and hyperglycemia. • No immediate feedback to the patient regarding the therapeutic or nutritional decision- less useful in preventing hypoglycemia* • Multiple comorbidities interfere with the values*^1^
	Rapid A1C assay (POC testing)	• Able to differentiate be^een pre-prandial and postprandial glycemia, possible hypo and hyperglycemia • Provides immediate feedback and allows timely therapy changes, more useful in preventing hypoglycemia*	• No information on daily glycaemic values • No information on daily glycaemic variability • Medical conditions could interfere with the values
Self-monitoring of blood glucose (before and after meals) (SMBG)	Finger-stick Glucose monitoring Glucometer with advanced features connected to smart devices (smartphone- enhanced glucometers)	• Provides real-time data and offers information on possible glycemic excursions • Allow patients to program their insulin to-carbohydrate ratio, correction factor, and target blood glucose level into the meter. • Offers information about the glycemic fluctuations • Take immediate therapeutic^2^ decisions/modifications	• Patient education/training is necessary • The elderly from Group II-IV may find difficulty independently using • SMBG • Need caregiver assistance* • The overall effect of SMBG on glyce- mic control is small • Cochrane Review 2012- Did not affect health-related quality of life or patient satisfaction [239]
Continuous Glucose Monitoring System (CGMS)	CGMS device	• Comprehensive image of the development of the disease and the treatment offers the ability to appreciate the glycemic tendency • Take immediate therapeutic decisions/modifications • Identify hypoglycemia, particularly the nocturnal and unawareness phenomena, and therefore may prevent associated falls and CV complications* • Useful in elderly persons using insulin* • Useful in elderly persons with physical or cognitive limitations who require monitoring of blood glucose by a surrogate*	• Not entirely identical to the glycemic level, certain lag • Patient education/training is necessary
Time in range	CGM Metric: Time spent in an individual's target glucose range (70-180 mg/dL)	• Simple and intuitive • Gives information regarding the qualify of glucose control • Extremely useful in elderly at risk of hypoglycemia unaware ness and complications* • Strongly correlated with A1C • Linked to the risk of developing vascular complications • Each incremental 5% increase in TIR is associated with clinically significant benefits [242]	
**Recommended level of Blood glucose**		**Required time**	
70-180 mg/dL <70 mg/dL >250 mg/dL		>50% (>12 h) <1% (<15 min) <10% (<2 h, 24 min)	

(Footnotes)

1 liver diseases, kidney diseases, hemoglobin disturbances, blood transfusions, pregnancy, hemolytic anemia, iron deficiency anemia, hypertriglyceridemia, hyperbilirubinemia, uremia, some drugs, alcohol abuse etc

2 pharmacological, nutritional, physical activity related, stress

A1C Recommendation: It should be performed twice annually for stable glycemic control and four times for unstable/need change in treatment [240].

Self-monitoring of blood glucose (SMBG): International guidelines recommend ≥ three times daily pre-prandial capillary blood glucose measurements for patients with intensive insulin therapy or insulin pump carriers; in other cases, the frequency of SMBG should be individualized depending on the treatment scheme and glycemic level control. Postprandial monitoring is recommended for cardiovascular prevention.

Continuous glucose monitoring-time in range (CGM-TIR): For older patients including high-risk ones (those with complications or co-morbidities, such as cardiovascular or kidney disease, cognitive deficits, osteoporosis, fractures, or joint disease), the TIR target is > 50% [241]. For the elderly TIR, the target was lowered from > 70% to > 50% and reduced TBR to < 1% at < 70 mg/dL (< 3.9 mmol/L) to place greater importance on reducing hypoglycemia than maintaining target glucose levels [242].

## 5 Technological Aids

### Methods to improve adherence and reduce dosing errors

Advances in technology are being used to improve elderly care and quality of life aiming at both disease-specific and elderly-specific parameters. **Table 14 and Table 15** The application of technological advances in elderly care may be broadly categorized.

**Table 14.** Aims of technological advances used in elderly with diabetes [243]

Disease-specific	Elderly-specific
• Control mechanisms for blood glucose (particularly hypoglycemia) • Cardiovascular risk factors • Prognosis monitoring • Specific nutritional recommendations (e.g., adequate-protein intake, daily dietary sodium intake < 2.3 g/day) • Suitable physical activity (e.g., exercise programs including aerobic and endurance training) • Education on diabetes • Self-care recommendations • Enhanced patient-healthcare communication tools	• Early detection of functional decline • Keep track of the functional changes to offer ways to encourage physical activity • Provide recommendations regarding adequate nutritional habits • Control polypharmacy

**Table 15.** Modules important for the management of elderly persons with diabetes [244]

Modules	Components	App Users
Geriatric assessment	Clinical tests, questionnaires	Clinicians
Preventive strategies	- Physical activity program through gamification - Nutritional recommendations - Pharmacotherapy plan	Elderly patient
Monitoring System	- Sensors to measure gait speed, strength in the lower limbs, involuntary weight loss, and level of physical activity - Processing of data provided by the monitoring system to detect deterioration alarms and initiate early measures to prevent disability	Clinicians/Caregivers
	- Treatment adherence - Other variables collected through questionnaires (e.g., recent falls, mental state, etc.)	Clinicians

Elderly persons have different barriers to adherence, the most common ones being remembering to inject themselves or injecting the correct dose or maintaining supplies. Therefore, in this patient population with diabetes, insulin pumps and continuous delivery devices certainly can facilitate improving adherence, and consequently, glycemic control [245]. Moreover, the use of an insulin delivery system is more accurate and precise than insulin administration via injections [249]. Use of insulin delivery systems also helps to reduce hypoglycemia and consequently its complications. **(Table 16)**

**Table 16.** Disease-specific technologies important for elderly persons with diabetes [244]

Technolog	Method	Application/Benefit
Insulin Delivery Systems	Pump or CSII	• Reduce hypoglycemia • Improve Alc • Availability of bolus calculators • Smaller accurate doses • Keep track of active insulin • Downloadable reports
	Wearable insulin delivery system	• A 24-hour patch delivers continuous stipulated basal insulin and on-demand bolus dosing by pushing a button at mealtimes • No alarm
	Inhaled insulin for prandial use	• Helpful for improving adherence, especially in patients who need lower doses of insulin at mealtimes.
	Bluetooth-enabled insulin pen	• Bolus calculator • For patients using multiple daily injections • Keep track of active insulin • Useful to assess adherence • Downloadable reports
Monitoring systems	CGM	• Alarm/alerts are available in most • Connected to smart devices like mobile phones, sharing data with caregivers is feasible • Reduce hypoglycemia • Reduce glucose variability • Improve glucose control • Reduce the need for fingersticks measurement • Downloadable reports
Hybrid closed-loop system	Combined glucose monitoring systems and insulin delivery systems	• A special feature that gets activated adjusts, suspends, and restarts insulin delivery based on the encoded glucose level. • Uses technolog to stop insulin delivery for up to 30 minutes to 2 hours if the glucose level reaches a preset low limit and the user doesn't react to a low-glucose alarm • Bolus prandial insulin needs to be initiated • Reduce hypoglycemia • Reduce glucose variability • Improve glucose control • Downloadable reports • Alarm/alert

Self-monitoring of blood glucose (SMBG) is a key component of diabetes management; however, it is limited by its staticity. Unlike SMBG, Continuous glucose monitoring (CGM) provides a trend of glycemic status, and therefore, is used for pattern management and assessment of glucose excursions. Hybrid systems combine insulin delivery and monitoring systems, enhancing the convenience of use [246,249]. The patients or the caregiver must be educated on interpreting and utilizing the information obtained from monitoring devices so that it affects adherence positively.

Self-monitoring is a basis of behavioral lifestyle interventions. The incorporation of personal fitness technologies into behavioral interventions enhances physical activity, decreases sedentary behaviours, and improves glycemic control in older adults. Activity trackers may boost physical activity through the amalgamation of analytically-tested behavioral change techniques such as goal-setting, self-monitoring, social support, social comparison, feedback, and rewards. Studies have observed weight loss, social connection, and increased activity awareness in the elderly using activity trackers **(Table 17 and Table 18)** [247,248].

**Table 17.** Technologies important for elderly persons with diabetes for Non-specific parameters [244]

Information and Communication Technologies (ICTs)	• Monitoring of vital signs • Provision of information through calls or educational contents	Monitors the evolution of gait speed, muscle power, and involuntar y weight loss to define preventive strategies to avoid disability.
Home-based technology mHealth technology (Apps in smartphones)	• May enable continuous and transparent monitoring of the independent elder	Identifies older people at risk of disability
Activity trackers – sensor-based wearable devices (smart watched, bands)	• Automatically track and monitor various indicators of physical activity - steps taken, stairs climbed, duration and quality of sleep, pulse or heart rate, calories consumed or burned, and even mood, moderate-intensity aerobic and muscle-strengthening activities - walking, jogging, bicycle riding, yard work, and gardening - Synchronize obtained data with users’ personal accounts, for easy access from any device.	• Convenient tool for this age group since it provides unbiased feedback about physical activity amount and intensity
	• Delivers prompts via short message ser vice	• Increases physical activity

**Table 18.** Diabetes technology based on the functional/health status of elderly persons [230]

Health status	Benefits		Requirement	Limitation
Healthy (few coexisting chronic illnesses, intact cognitive and functional status) **Group I**	Bluetooth pen: Can be used to keep track of adherence and educate patients regarding the impact of missed or inaccurate dosing Pump: • Capacity for a small dose of insulin • Assistance with insulin calculator and active insulin on board • Provide flexibility CGM: • Reduced need for finger sticks • Alarm and alert can help with hypoglycemia fear and unawareness • Features for sharing can be used to involve caregivers as needed		• Need to evaluate cognitive function periodically • Patients and Caregivers need to be trained to utilise the information obtained	• Alarms and alert fatigue can cause anxiety
Patients receiving care in a skilled nursing facility for short-term rehabilitation **Group II**	Pump: • May maintain tighter control needed during rehabilitation CGM: • Can help lower the risk of hypoglycemia especially if on an insulin regimen		• Staff Training	
Very complex/poor health (long-term care or end- stage chronic illnesses or moderate-to-severe cognitive impairment or 21 ADL dependencies) **Group III**	Pump: • Consider continuing pump in older adults if staff can support • CGM: • Continue CGM therapy to prevent unrecognized hypoglycemia episodes (hypoglycemia unawareness) in those on multiple insulin injections or those who are not tolerating fingerstick		• Staff Training	
Patients at end of life **Group IV**	Avoid extremes of glucose level		• Not much role	

### Special Situations

#### Religious/Cultural Fasting

Many patients with diabetes may fast for religious or cultural reasons. Elderly persons often are more religious and may insist on fasting. Fasting could be either regular or intermittent for a few days in a week or continuously for an entire month. Management of intermittent fasting is more challenging because the pharmacotherapy schedule becomes more complex compared to when it is continuous. Maintaining euglycemic status is even more complex in patients using insulin therapy. The rate of complications is significantly greater in older adults than in younger ones with diabetes. Therefore, management is even more challenging for the elderly. Besides, this population is excluded in most trials, and guidance is based on expert opinions rather than clinical evidence.

The experts suggest the management of diabetes should be attuned to the patient’s lifestyle, not the opposite as a reassurance of living a normal life even with diabetes. For the elderly who wish to fast, a careful risk-benefit analysis must be performed. The results should be discussed with the person and his/her family to arrive at a shared decision.

The other aspect of fasting is dehydration in case liquids are also restricted or the patient is on diuretics or SGLT-2i or those with brittle diabetes or renally impaired. Most liquid intake depends on the type of food consumed, particularly in the elderly who depend on a liquid diet (juices and soups). These patients also should avoid fasting or can observe fasting provided extreme caution is followed along with recommended dietary and medication modifications. If the patient still insists on fasting, the use of SGLT2i must be avoided.

#### Sick Day Rule

Most common illnesses in the general population can assume serious complications in people with diabetes, particularly in the elderly who have a compromised immune system. Certain illnesses like common cold or flu, upper and lower respiratory infections, urinary tract infections gastrointestinal tract infections, and skin infections are accompanied by high body temperature. These illnesses can dysregulate the plasma glucose resulting in serious complications like DKA or HHS.

Our experts agree with the ADA and IDF-Europe recommendations for the management of sick patients with diabetes. The anti-diabetic medication should be continued; in particular, insulin should not be discontinued but plasma glucose should be more frequently monitored (at least every four hours). Adequate hydration and oral caloric intake should be maintained [250,251]. Glucose lowering therapy should be adjusted according to oral caloric intake.

Fatigue, weight loss, and polyuria are indicators of hyperglycemia. Plasma glucose above 270 mg/dL, ketones in the urine, and excessive thirst are signs of DKA. Vomiting, abdominal pain, tachycardia, and reduced level of consciousness constitute medical emergencies.

#### Inpatient management

In critically ill patients in the ICU as well as for most patients admitted for general illnesses or surgery in the non-ICU setting, the various guidelines recommend target glucose between 140-180 mg/dl **(Table 19)**.

**Table 19.** Major guidelines for treatment of hyperglycemia in a hospital setting

	ICU	Non-ICU
ADA/AACE	Initiate insulin therapy for persistent hyperglycemia (>180 mg/dl) Treatment goal: 140 - 180 mg/dl if achievable without significant risk for hypoglycemia.	No specific guidelines. If treated with insulin, pre-meal glucose targets should generally be <140 mg/dl, with RBG levels <180 mg/dl Less stringent targets may be appropriate for people with severe comorbidities.
ACP	Recommends against intensive insulin therapy in surgical/medical ICUs Treatment goal 140 - 200 mg/dl, in surgical / medical ICUs	
Critical Care Society	>150 mg/dl should trigger insulin therapy Treatment goal: <150 mg/dl in ICU. Maintain glucose levels <180 mg/dl while avoiding hypoglycemia.	
Endocrine Society		Pre-meal glucose target <140 mg/dl and RBG <180 mg/ dl.
		Terminal illness and/or with limited life expectancy or at high risk for hypoglycemia <180 - 200 mg/dl Adjust antidiabetic therapy when the glucose falls <100 mg/dl to avoid hypoglycemia.
Society of Thoracic Surgeons (Guidelines specific to adult cardiac surgery)	Continuous insulin infusion preferred over SC or intermittent intravenous boluses Treatment goal: <180 mg/dl during surgery (≤110 mg/dl in fasting and pre-meal states)	
Joint British Diabetes Society for Inpatient Care		108 - 180 mg/dl with an acceptable range of between 72 - 216 mg/dl

AACE/ADA, American Association of Endocrinologists and American Diabetes Association joint guidelines; ACP, American College of Physicians; ADA, American Diabetes Association; ICU, intensive care unit

Insulin, preferably delivered intravenously, remains the best alternative for maintaining glycemic status in the inpatient setting, specifically in the ICU. However, evolving data suggest that OHAs from the DPP-4i class either as monotherapy or in combination with basal insulin may be used in patients with mild to moderate hyperglycemia. In a non-ICU setting (general medicine or surgery) the recommended total daily insulin dose for most people should start between 0.15-0.3 units/kg body weight, with lower doses of 0.1-0.15 units/kg recommended for older patients with renal failure (eGFR < 60 mL/min/1.73 m^2^), history of hypoglycemia, or poor oral intake [252]. In addition, patients with enteral or parenteral nutrition should be monitored for glucose at 4-6-hour intervals to avoid hypo-hyperglycaemia [171].

## 6 Summary

The panel opined that the current and expected geriatric population with type 2 diabetes in Asia is enormous along with a huge spectrum of dietary and disease (communicable and non-communicable) disparities unique to the region. Hence, management of diabetes in the Asian geriatric population is indeed challenging. The Best Practice Document has considered the conventional as well as unique factors when proposing recommendations for glycemic targets. Particular attention has been paid to the geriatric syndrome and management of type 2 diabetes with pharmacological and non-pharmacological interventions aligned to its existence. The use of technological aids to monitor the disease status and avoid several risks specifically in this vulnerable population has been emphasized. Modifications to diabetes management in special situations like religious fasting or hospitalization or days when they feel unwell have been addressed. The panel has been thorough in addressing aspects specific to geriatric diabetes.

## Abbreviations

2hG: 2-hour glucose AACE American Association of Clinical Endocrinology

ACCORD: Action to Control Cardiovascular Risk in Diabetes

AD: Alzheimer’s disease

ADA: American Diabetes Association

ADVANCE: Action in diabetes and vascular disease: preterax and diamicron mr controlled evaluation

AEDs: Anti-epileptic drugs

AGA: American Geriatrics Association

AGE: Advanced glycation end products

AWGS: Asian Working Group for Sarcopenia

BBB: Blood Brain Barrier

BLSA: Baltimore Longitudinal Study of Aging

BMD: Bone mineral density

BMI: Body mass index

CBT: Cognitive Behavioral Therapy

CGM-TIR: Continuous glucose monitoring-time in range

CHD: Coronary heart disease

CKD: Chronic kidney disease

CVD: Cardiovascular disease

DPN: Diabetic peripheral neuropathy

EASD: European Association for the Study of Diabetes

ED: Emergency department

ESE: European Society of Endocrinology

FPG: Fasting plasma glucose

GIP: Glucose-dependent insulinotropic polypeptide

GLP-1: Glucagon-like peptide-1

GSA: Gerontological Society of America

HHS: Hyperglycemic hyperosmolar syndrome

HRT: Hormone replacement therapy

ICU: Intensive care unit

IDF: International Diabetes Federation

ISBMR: Indian Society for Bone and Mineral Research

IU: international units

LDL: Low-density lipoprotein LOH Late-onset hypogonadism

MCI: Mild cognitive impairment MNA Mini Nutritional Assessment

NHANES: National Health and Nutrition Examination Survey

NMT: Not more than

NPI: Non-pharmacological Interventions

OGTT: Oral glucose tolerance test

PPBG: Post prandial blood glucose

PVD: Peripheral vascular disease

QoL: Quality of life

SD: Standard Deviation

SGLT-2i: Sodium-glucose Cotransporter-2 Inhibitors

SIADH: Syndrome of inappropriate antidiuretic hormone

SMBG: Self-monitoring of blood glucose

SNRI: Serotonin and norepinephrine reuptake inhibitors

SSRI: Serotonin and norepinephrine reuptake inhibitors

TBR: Time below range TIMES2 Testosterone replacement In hypogonadal men with either metabolic Syndrome or type 2 diabetes

TOS: The Obesity Society

TRT: Testosterone replacement therapy

TZD: Thiazolidinediones

USA: United States of America

USPSTF: United States Preventive Services Task Force

UTI: Urinary tract infections

VADT: Glycemic Control and Complications in Diabetes Mellitus Type 2

VMS: Vasomotor symptoms

WHO: World Health Organization
